# Broken replication forks trigger heritable DNA breaks in the terminus of a circular chromosome

**DOI:** 10.1371/journal.pgen.1007256

**Published:** 2018-03-09

**Authors:** Anurag Kumar Sinha, Christophe Possoz, Adeline Durand, Jean-Michel Desfontaines, François-Xavier Barre, David R. F. Leach, Bénédicte Michel

**Affiliations:** 1 Bacterial DNA stability, Genome biology department, Institute for Integrative Biology of the Cell (I2BC), CEA, CNRS, Université Paris-Sud, Université Paris-Saclay, Gif-sur-Yvette, France; 2 Evolution and maintenance of circular chromosomes, Genome biology department, Institute for Integrative Biology of the Cell (I2BC), CEA, CNRS, Université Paris-Sud, Université Paris-Saclay, Gif-sur-Yvette, France; 3 Institute of Cell Biology, School of Biological Sciences, University of Edinburgh, Edinburgh, United Kingdom; University of Washington School of Medicine, UNITED STATES

## Abstract

It was recently reported that the *recBC* mutants of *Escherichia coli*, deficient for DNA double-strand break (DSB) repair, have a decreased copy number of their terminus region. We previously showed that this deficit resulted from DNA loss after post-replicative breakage of one of the two sister-chromosome termini at cell division. A viable cell and a dead cell devoid of terminus region were thus produced and, intriguingly, the reaction was transmitted to the following generations. Using genome marker frequency profiling and observation by microscopy of specific DNA loci within the terminus, we reveal here the origin of this phenomenon. We observed that terminus DNA loss was reduced in a *recA* mutant by the double-strand DNA degradation activity of RecBCD. The terminus-less cell produced at the first cell division was less prone to divide than the one produced at the next generation. DNA loss was not heritable if the chromosome was linearized in the terminus and occurred at chromosome termini that were unable to segregate after replication. We propose that in a *recB* mutant replication fork breakage results in the persistence of a linear DNA tail attached to a circular chromosome. Segregation of the linear and circular parts of this “σ-replicating chromosome” causes terminus DNA breakage during cell division. One daughter cell inherits a truncated linear chromosome and is not viable. The other inherits a circular chromosome attached to a linear tail ending in the chromosome terminus. Replication extends this tail, while degradation of its extremity results in terminus DNA loss. Repeated generation and segregation of new σ-replicating chromosomes explains the heritability of post-replicative breakage. Our results allow us to determine that in *E*. *coli* at each generation, 18% of cells are subject to replication fork breakage at dispersed, potentially random, chromosomal locations.

## Author summary

The *Escherichia coli recBC* mutant, deficient for DNA double-strand break (DSB) repair, shows a viability defect and a specific deficit in the level of chromosome terminus DNA sequences. We previously showed that this deficit results from heritable terminus DNA loss, owing to cell-division dependent DSBs in the chromosome terminus. Here, we used whole genome sequencing and microscopy to analyse the phenomenon. Our results allow us to conclude that in *E*. *coli* most spontaneous DSBs occur at replication forks, and that such breaks occur in 18% of cells at each generation. In a *recBC* mutant the linear chromosome arm made by replication fork breakage is neither repaired nor degraded. Thus it remains attached to the circular chromosome part, which triggers a DSB in the chromosome terminus during cell division in a heritable reaction. In wild-type cells, broken replication forks are repaired and these terminus DSBs do not occur. Our study reconciles the idea that replication fork impairment is a major source of chromosome breakage with the observation that most DSBs in a *recBC* mutant occur in the chromosome terminus during cell-division and reveals the links between these two phenomena.

## Introduction

**Fig 1:**
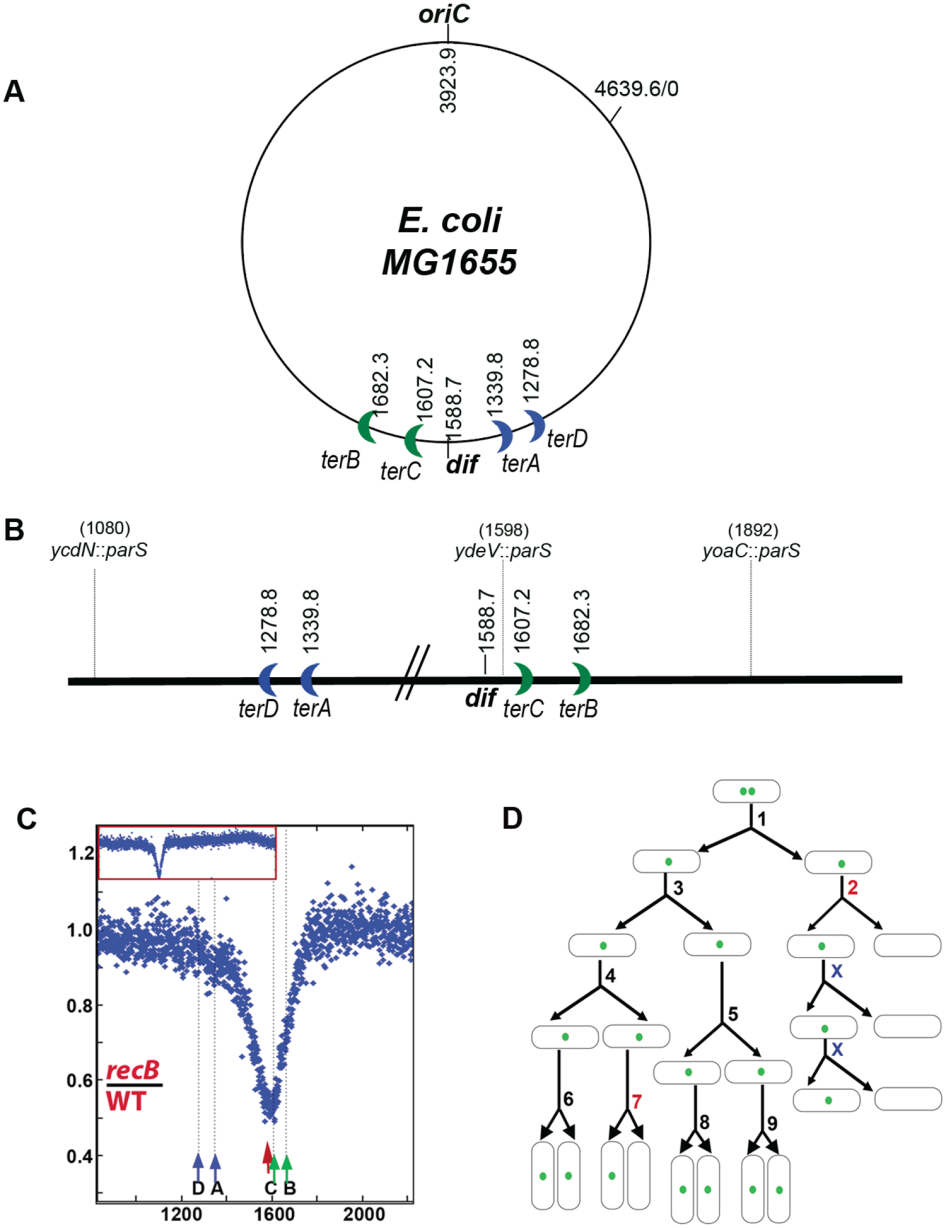
(A) Circular map of the *E*. *coli* chromosome: *oriC*, *dif* and *terD* to *terB* sites are indicated. Numbers refer to the chromosome coordinates (in kb) of MG1655. (B) Linear map of the terminus region: chromosome coordinates are shown increasing from left to right, as in the marker frequency panels (see Figure 1C for example), therefore in the opposite direction to the circular map. In addition to *dif* and *ter* sites, the positions of the *parS*_pMT1_ sites used for microscopy experiments are indicated. (C) MFA analysis of terminus DNA loss in the *recB* mutant: sequence read frequencies of exponential phase cells normalized to the total number of reads were calculated for each strain. Ratios of normalized reads in isogenic wild-type and *recB* mutant are plotted against chromosomal coordinates (in kb). The profile ratio of the terminus region is enlarged and the profile of the corresponding entire chromosomes is shown in inset. Original normalized profiles used to calculate ratios are shown in S1 Fig. The position of *dif* is indicated by a red arrow. The *ter* sites that arrest clockwise forks (*terC*, *terB*, green arrow) and counter-clockwise forks (*terA*, *terD*, blue arrow) are shown. (D) Schematic representation of focus loss in the *recB* mutant: Time-lapse microscopy experiments showed that loss of a focus in the *recB* mutant occurs concomitantly with cell division in one of two daughter cells, and that the cell that keeps the focus then generates a focus-less cell at each generation. The percentage of initial events was calculated as the percentage of cell divisions that generate a focus-less cell, not counting the following generations. In this schematic representation, two initial events occurred (generations #2 and #7) out of 9 generations, and focus loss at generation #2 is heritable. Panels shown in this figure were previously published in [19] and are reproduced here to introduce the phenomenon.

The bidirectional replication of the *Escherichia coli* circular chromosome starts at the replication origin *oriC* and ends when forks meet in the opposite region, the chromosome terminus. Replication forks are arrested in the terminus region by specific sites called *ter* where binding of the Tus protein blocks replication forks in an orientation-specific manner (reviewed in [1,2]). *ter* sites are oriented to form a replication fork trap, replication forks can enter the trap but their exit is delayed by pauses at several successive *ter* sites (Fig 1A and 1B). As chromosome segregation is concurrent with replication in bacteria, the origin and terminus regions are also the first and the last DNA sequences to be segregated during chromosome partitioning [3–5]. Following replication initiation, the two origins first remain associated at mid-cell for about 20 min and then move to the ¼ and ¾ positions of the cell. Then, the chromosome arms segregate from mid-cell to these positions as they are replicated. Finally, the terminus regions are also replicated at mid-cell and only separate shortly before cell division [3–5].

The chromosome terminus is organized in a large Ter macrodomain of about 780 kilobases (kb) by binding of the MatP protein to specific DNA motifs, the *matS* sites [6]. MatP also interacts with the septum protein ZapB, and thus maintains the Ter macrodomain at midcell during septum formation [7–9]. The terminus region is centred on a specific site called *dif*, the target of recombinases XerC and XerD for chromosome dimer resolution (reviewed in [10,11]). *dif* is positioned opposite *oriC* on the circular chromosome (Fig 1A), and is the inversion point of the GC strand skew. Specific motifs, KOPS (FtsK oriented polar sequences), which provide directionality of chromosome segregation, converge at the *dif* site (reviewed in [12]). They are recognized by the C-terminal domain of a septum-protein, the FtsK translocase which acts as an oriented DNA pump. KOPS motifs point from the origin of replication towards *dif*, allowing FtsK to bring newly replicated *dif* sites together at mid-cell and to remove DNA from the constricting septum [13,14]. As a result *dif* sites are the last region to be segregated away from mid-cell [5,15]. Recently a new phenomenon was described in the terminus region. Sequencing of the entire genome and analysis of DNA sequence coverage as a function of position on the chromosome (Marker Frequency Analysis, MFA) has revealed a deficit of sequences in the chromosome terminus region in the *recB* mutant [16–18]

DNA double strand break (DSB) repair in *E*. *coli* is entirely dependent on homologous recombination, first steps of which are catalysed by RecBCD and RecA (reviewed in [20–22]). RecBCD is a heterotrimeric complex that binds to double-stranded DNA (dsDNA) ends. RecB and RecD are helicases, and RecB also acts as a nuclease. RecBCD degrades dsDNA ends until it encounters specific DNA motifs called *chi* sites, after which it continues to degrade the 5’ end. It then loads RecA on the protruding 3’ tail for homology search, strand invasion and strand exchange. The resulting Holiday junctions are resolved by RuvABC resolvase to generate recombination products. In the absence of RecA, DSBs lead to chromosome degradation because of the potent exonuclease activity of RecBCD. Indeed the complex was originally characterised as the major *E*. *coli* exonuclease, Exo V. *recB* and *recC* null mutants are deficient for DSB repair, but because the RecBC complex can still catalyze strand opening and RecA loading, *recD* mutants are Rec^+^. However, Exo V activity is abolished in all three null mutants, *recB*, *recC* and *recD*, even though the *recD* mutants still degrade linear DNA *in vivo* at 50% of the wild-type rate [23] Finally, RecBCD-dependent homologous recombination is coupled with replication restart, which allows chromosome replication to resume after the repair by homologous recombination of broken replication forks (reviewed in [24]).

In a previous study we showed that the deficit of terminus DNA sequences observed in the chromosome of *recB* mutant cells, which we call terminus DNA loss (Fig 1C, S1 Fig), was independent of all known DNA processing events to take place in the terminus: replication fork merging, dimer resolution and decatenation of the two circular replicated chromosomes [19]. It also occurred in cells lacking FtsK-mediated chromosome segregation, but in an *ftsK* mutant, terminus DNA loss became less centred at *dif*, indicating a role for FtsK in the positioning of the peak of DNA loss around the site of convergence of KOPS sequences [19]. Our study led to the following key observations: (i) terminus DNA loss occurred during septum closure and required cell division, (ii) a first cell division generated one daughter cell that lacked the terminus sequence, and one that retained it (the initial event), (iii) the daughter cell that carried the terminus sequence generated again a non-proliferating terminus-less cell and a viable terminus-containing cell, at each following generation (heritable, transmitted events; [19]; Fig 1D). Furthermore, our analysis by RecA ChIP suggested that these terminus DSBs did not occur in wild-type cells, and were thus caused by the absence of RecBCD [19]. Here we have taken forward our previous study and used MFA and cell biology techniques to understand these mysterious observations. We propose and test a model in which, in a *recB* mutant, replication fork breakage triggers a terminus DSB during cell division in a heritable manner. Our results allow us to conclude that in wild-type, untreated *E*. *coli* cells, chromosome DSBs occur mainly at replication forks, and to determine the frequency of spontaneous replication fork breakage to be ~18% per cell per generation.

## Results

### A model for cell division-induced terminus DNA loss

We studied terminus DNA loss by a combination of MFA and microscopy analyses. For microscopy, we used strains that constitutively express the yGFP-ParB_pMT1_ fusion protein from a chromosome-inserted gene and carry a *parS*_pMT1_ site at one of three different loci (Fig 1B). Binding of yGFP-ParB_pMT1_ to its cognate recognition site allows the visualization of each *parS* sequence as a fluorescent focus [25]. Three different strains were used, which carry *ydeV*::*parS*_pMT1_ between *dif* and *terC*, 10 kb from each, or *yoaC*::*parS*_pMT1_ about 300 kb away from *dif* on the left replichore, or *ycdN*::*parS*_pMT1_ about 500 kb away from *dif* on the right replichore [19] (Fig 1B, S1 Table). All experiments were carried out in M9 glucose medium (called M9 henceforth). Exponentially growing wild-type cells showed one or two foci. Cells with two foci depended on whether the *parS*_pMT1_ site was replicated and segregated and therefore decreased with distance of the site from the origin [25] (S2 Table). In a *recB* mutant ~30% of cells showed no *dif*-proximal focus (*ydeV*::parS_pMT1_), and ~7–8% showed no *dif*-distal focus (*yoaC*::*parS*_pMT1_, *ycdN*::*parS*_pMT1_) [19] (Table 1, S2 Table). Time-lapse microscopy experiments allowed the real time visualization of focus loss in *recB* mutant cells: ~18% of the divisions produced a focus-less cell and a daughter cell with a focus [19] (“% initial events” in Table 1; S1 Video) and focus loss was heritable in ~75% of the cases [19] (Fig 1D; “% transmitted” in Table 1; S1 Video; these inherited events are not counted in the 18% initial events).

**Table 1:** Terminus DNA loss in recombination mutants.

genotype	% cells with 0 focus^(a)^		*ydeV*::*parS*_pMT1_	
	*ydeV*::*parS*_pMT1_	*yoaC*::*parS*_pMT1_	initial events^(a)^	transmitted^(a)^
*wild-type*^(b)^	0.6 ± 0.2	0.6 ± 0.3		
*recB*^(b)^	32 ± 1.5	7.9 ± 1	17.7% (350)	74.5%
*recA*	9 ± 2.8	8.8 ± 0.9	7.0% (1416)	37.2%
*recD*	0.6 ± 0.7	0.34 ± 0.37		
*recA recB*	36.6 ± 1.5	8.5 ± 1.6	21% (362)	83.7%
*recA recD*	27.3 ± 2.1	23 ± 2.1	16.1% (242)	65%
*sbcB sbcD*	1 ± 0.1	1.7 ± 0.8		
*sbcB sbcD recA*	31 ± 1.2	11.6 ± 1	19.8% (511)	68%
*recA sbcB*	16.7 ± 4.6		12.1% (605)	48.8%
*recA sbcD*	15.3 ± 1.6			
*sbcB sbcD recB*	29.6 ± 2.2	5.9 ± 0.2	9.8% (471)	27.3%
*ruvAB*	6.8 ± 1.1			
*ruvAB recB*	37 ± 2.1			
*ruvAB recA*	11.7 ± 0.7			
*ruvAB recA recB*	37.6 ± 2.2		20.7% (463)	60%
*recA tus*	16.5 ± 0.9		11.2% (626)	64.1%
*matP*	1 ± 0.9	1.4 ± 0.06		
*matP recB*	37.6 ± 2	9.2 ± 1.5	15.5% (453)	86.4%
*ftsK*^*ΔCter*^^(b)^	25.1 ± 1.9	4.5 ± 2.3		
*ftsK*^*ΔCter*^ *recB*^(b)^	54.4 ± 1.2	15.9 ± 3.1	15.8% (303)	82.8%
*ftsK*^*ΔCter*^ *matP*^(c)^	14.6 ± 2.1			
*ftsK*^*ΔCter*^ *matP recB*	39.7 ± 1.2			

^(a)^ In all tables, “% cells with 0 focus” are averages from two or three independent snapshot experiments ± standard deviations (see S2 Table for the number of experiments and the total number of cells analysed). Initial events and percentage of transmitted events were calculated by summing the results of two or three independent time-lapse experiments. The numbers between parentheses indicate the total number of generations analysed.

^(b)^ Published in [19]. In all *ftsK*^*ΔCter*^ mutants, about 15% focus-less cells result from the lack of dimer resolution and “guillotining” of chromosome dimers.

We observe here that 10–15% additional focus-less cells result from the presence of MatP.

^(c)^ A high proportion of cells are elongated

**Fig 2:**
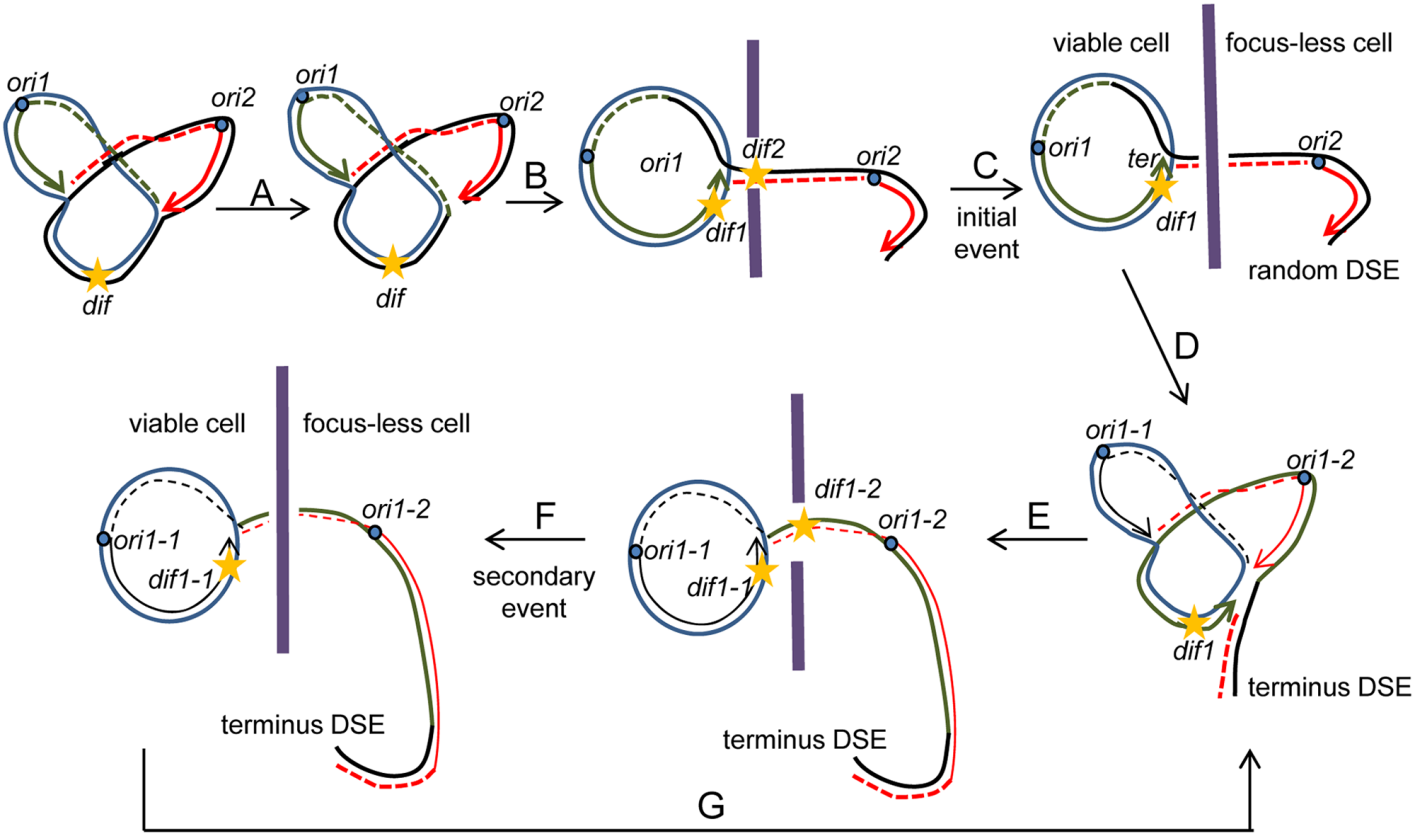
Model for terminus DNA loss in the *E*. *coli recB* mutant by formation of a σ-replicating chromosome. A) In the first step, one chromosome arm is broken at a replication fork. In the example shown, this random initial DSB occurs on the clockwise replication fork, but the reaction is entirely symmetrical and breakage of the other replication fork can also form a σ-replicating chromosome with a tail ending at this first DSB random position. In a wild-type strain the broken chromosome arm is repaired by RecBCD- RecA-mediated homologous recombination (not drawn). In a *recB* mutant the DNA end is slowly degraded by the combined action of helicases and ssDNA exonucleases. In the example shown, the leading strand template is broken (or was interrupted prior to arrival of the replication fork), and the parental strand (black line) is linked to the lagging strand at the fork (green dashed line) by gap filling and ligation. The position of the *ydeV*::*parS*_pMT1_ focus next to *dif* is indicated by a yellow star. B) The intact replication fork progresses toward the terminus while the broken chromosome arm, which carries a replication origin, segregates to the other cell half and is separated from the intact homologous sequence by septum formation. The *ydeV*::*parS*_pMT1_ locus next to *dif* is duplicated. (C) At cell division, the linear arm in the terminus region is broken during cell division; in the presence of FtsK the septum closes on the KOPS convergence point, *dif*. Note that since the induction of the SOS response by dsDNA ends requires RecBCD, division is not prevented by the SOS-induced SfiA protein in a *recB* mutant. Septum closure is concomitant with the disappearance of the *ydeV*::*parS*_pMT1_ focus from one daughter cell. The two dsDNA ends created by septum closure are slowly degraded, generating the first focus-less cell that contains a partial chromosome. The cell that shows a focus carries a circular sigma-replicating chromosome with a shortened tail, and an intact fork from the first replication round, which is slowed down by *ter* sites. D) After cell division, a new replication round is initiated. E) The first counter-clockwise replication fork and the new clockwise fork merge. The strands made by copying the intact circular strand (dashed blue and green lines, copies of the blue line) are linked to produce the circular part of a σ-replicating chromosome. The strands made by copying the linear part (dashed and full red lines, copies of the black-green line) are linked to produce a tail containing an entire chromosome. The enlarged tail carries a replication origin, it segregates to the other half of the cell. F) Septum closure cleaves the tail DNA in the terminus region, producing a σ-replicating chromosome as in step C and the second focus-less, originally containing a nearly full linear chromosome in which the terminus DNA sequences are slowly degraded. G) The σ-replicating chromosome with a short tail originally interrupted at *dif* is replicated. More cycles of replication-breakage events (steps E-F-G) will generate a focus-less cell at each generation and reset the tail length on the sigma-replicating chromosome to the distance between the *dif* site and the position of the intact fork at each cell division. Blue and black thick lines, original chromosome strands; red and green thick lines, DNA synthesized at the first generation; black and red thin lines, DNA synthesized at the second generation; purple thick line, septum; full lines represent leading-strands and dashed lines lagging-strands, arrows indicate the 3’ DNA ends; the positions of origins (*ori*, blue small circles) and *dif* sites are indicated; the position of the *ydeV*::*parS*_pMT1_ locus is shown with a yellow star.

**Fig 3:**
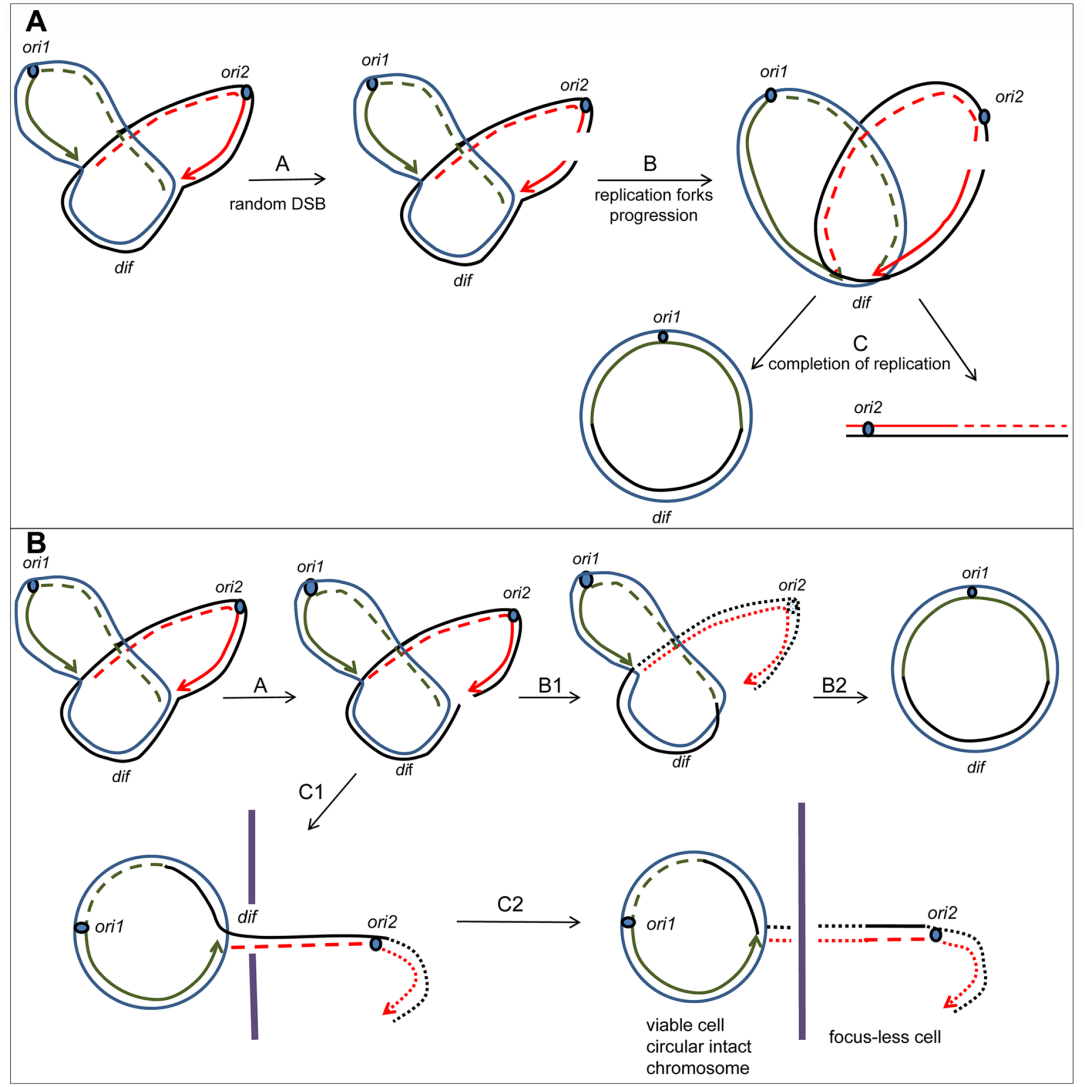
Only fork breakage accounts for heritable terminus DNA loss. A. In a *recB* mutant, a random DSB in the replicated region is not repaired (A), but both replication forks can progress (B), until they merge in the terminus region and produce one intact chromosome and one linear chromosome interrupted at the position of the initial DSB (C). The slowly degraded dsDNA ends are not at *dif* and form independently of cell division. Blue and black thick lines, original chromosome strands; red and green thick lines, DNA synthesized at the first generation; full lines represent leading-strands and large dashed lines lagging-strands, narrow dashed lines represent degraded DNA, arrows indicate the 3’ DNA ends; the position of origins (ori, blue small circles) and *dif* sites is indicated. B. In a *recA* mutant, degradation of linear DNA by RecBCD limits terminus DNA loss. (Step A) in the *recA* mutant the reaction also starts by replication fork breakage. Pathway B: (B1) the dsDNA end is bound by RecBCD which entirely degrades the linear part of the σ-replicating chromosome. (B2) this DNA degradation produces an intact circular chromosome, and no focus-less cell is formed. Pathway C: (C1) the dsDNA end is not degraded prior to segregation and the septum closes on the tail *dif* site. (C2) the terminus DNA is cleaved by septum closure. In the focus-containing cell, degradation by RecBCD of the short tail produces a circular chromosome and prevents heredity. In the focus-less daughter cell, the linear chromosome will ultimately be fully degraded by RecBCD to produce an anucleate cell.

The molecular model depicted in Fig 2 explains these observations and has been tested in the present work. The model is as follows: a dsDNA end formed by breakage of one replication fork, at a dispersed and potentially random chromosomal location, results in a structure called a σ-replicating chromosome. This consists of an entire circular chromosome covalently linked to a linear partial chromosome arm by one intact replication fork (Fig 2, step A). The linear arm is repaired by homologous recombination in wild-type cells, but remains unrepaired in a *recB* mutant, in which σ-replicating chromosomes have been proposed to prevent cell growth [26,27]. We propose that in a *recB* mutant the linear and circular parts of this σ-replicating chromosome segregate to the two halves of the cell, while the intact replication fork progresses toward the terminus, and pauses at the *ter* sites (Fig 2, step B). However, the linear arm of the σ-replicating structure necessarily passes through mid-cell and is processed by FtsK, which precisely positions *dif* in the constricting septum (Fig 2, step C). The trapped DNA is broken during cell division, producing one daughter cell containing a linear, partial chromosome (focus-less cell) and the other one containing a σ-replicating chromosome with a shortened tail (Fig 2, step C). The DNA ends made during septum closure are located near *dif* and are slowly degraded by exonucleases. A second round of replication is initiated at *oriC* (Fig 2, step D) and the tail of the σ-replicating chromosome is enlarged by the entire newly replicated sequence when the intact replication fork of the σ-replicating chromosome merges with the fork of the second replication round (Fig 2, step E). This new σ-replicating chromosome contains a complete linear chromosome attached to the terminus of a circular chromosome. The circular and linear parts segregate to daughter cells, and the region around the *dif* site, maintained in the path of the septum by the FtsK translocase, is cleaved again during cell division (Fig 2 step F). This accounts for the efficient transmission of the phenomenon to the progeny in *recBC* mutants, as terminus breakage creates again a circular chromosome with a short tail and therefore the cycle of events can resume (Fig 2, step G). Importantly, we propose here that the initial DSB occurs at a replication fork, because a DSB elsewhere in the replicated region would leave both forks intact (Fig 3A). Replication would produce a circular chromosome with no scar and a linear chromosome interrupted at a random sequence, which cannot account for our observations of heritable terminus DNA loss during division and DNA degradation centred on *dif*.

### Terminus DNA loss is less efficient in a *recA* mutant than in a *recB* mutant

**Fig 4:**
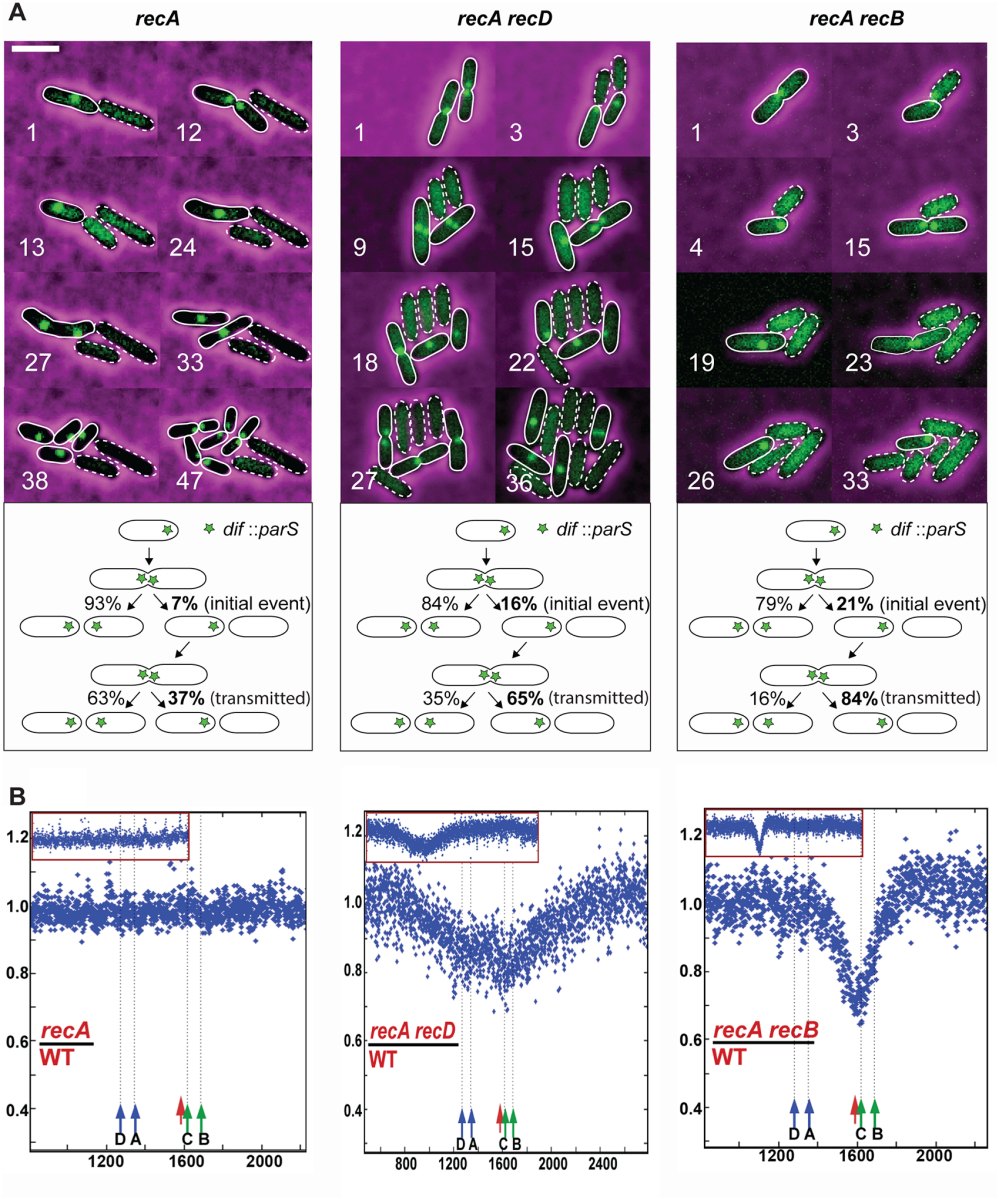
Terminus DNA loss in *recA* mutants. (A) Time-lapse analysis of focus loss in *recA* (left panel), *recA recD* (middle panel) and *recA recB* (right panel) mutants. Time-lapse experiments were carried out on M9 glucose agarose pads at 30°C with pictures taken every 10 min. Cells contain *ydeV*::*parS*_pMT1_ and express the ParB_pMT1_ protein from the gene inserted into the chromosome. The numbers in the lower left corner of the pictures indicate the frame number. For reasons of space limitations some frames are skipped. Cells that generate a focus-less cell during division are circled with a full white line. Most often two foci can be seen before division, which shows that focus loss results from the degradation of a DNA sequence that has been previously replicated. Cells that have lost the focus are circled with a dashed white line. These focus-less cells generally do not divide. In the *recA* mutant example (left), focus loss is transmitted for one generation (images number 1 and 13) and then the focus-carrying cell returns to normal divisions (images 33–47). In the *recA recD* mutant transmission is increased compared to the *recA* mutant, two examples are shown. The cell on the left generates a focus-less cell at each cell division for 3 generations (transmitted event, images number 3, 15, 22) before returning to a normal division (images 27–36). The cell on the right generates a focus-less cell (image 3) and then divides normally once (images 15–18, non-heritable event). At the next generation each focus-containing cell undergoes a new initial event (image 36); these late initial events were counted but not used to quantify heredity since the following generations were not visible. In the *recA recB* example (right), a focus-less cell is generated during 5 consecutive generations. Examples of focus-less cell production from a cropped bacterium, but for which all frames taken every 10 min are shown, can be seen in S1 Video (*recB*), S2 Video (*recA*), S3 Video (*recA* elongated cells) and S4 Video (*recA recD*). A schematic representation showing the frequency of initial and heritable events is shown below the time-lapse images. (B) MFA analysis of terminus DNA loss in the *recA* (left panel), *recA recD* (middle panel) and *recA recB* (right panel) mutants. Experiments are realized and plotted as in Fig 1C. Original MFA data are shown in S2 Fig.

In a *recA* mutant, dsDNA ends are acted upon by RecBCD and linear DNA is very efficiently degraded. We predicted that both the first linear tail created by fork breakage and the second, smaller linear tail created by division-induced breakage should be degraded by RecBCD in *recA* cells, reducing initial events and transmission of the phenomenon, respectively (Fig 3B). We observed that the percentage of focus-less cells was three-fold lower in the *recA* mutant (9%) than in the *recB* mutant (~32%, Table 1, S2 Table). Time-lapse experiments showed that focus loss occurred in *recA* cells with some of the characteristics of *recB* cells: it occurred most frequently at the septum, always at the time of cell division and in one daughter cell only (Fig 4A left panel; complete movie is shown in S2 Video). However, the proportion of initial events in the *recA* mutant was 7% of total divisions, nearly three-fold less than in the *recB* mutant (17.7%, Table 1, Fig 4A left panel). Furthermore, transmission of the phenomenon to progeny was less efficient in the *recA* than in the *recB* mutant, since (i) ~37% of events were transmitted to progeny instead of ~75% in *recB* cells, and (ii) the number of successive generations undergoing terminus DNA loss was reduced compared to the *recB* mutant: for example, among the events that could be followed for more than 3 generations, 19 out of 27 continued focus loss in the *recB* mutant *versus* only 2 out of 12 in the *recA* mutant, the other ones mostly returning to normal growth. Note that the percentage of heritable events decreased from 13.3% of all divisions in the *recB* mutant (75% of 17.7% of the divisions) to 2.6% in the *recA* mutant (37% of 7% of the divisions). Furthermore, 5–10% of divisions in the *recA* mutant were preceded by cell elongation, and some elongated cells produced focus-less cells (S3 Video). This cell elongation could result from a partial degradation of the long DNA tail, which might prevent a correct DNA segregation and, in turn, block septum formation until the following replication round.

In addition, in *recA* mutant cells we observed a similar percentage of cells lacking the *dif*-proximal *ydeV*::*parS*_pMT1_ locus and the *yoaC*::*parS*_pMT1_ locus further from *dif* (~9%; Table 1; S2 Table), and no terminus DNA loss could be detected by MFA ([17]; Fig 4B left panel, S2 Fig). The *recA* mutants are known to lose entire nucleoids, and ~10% loss of terminus corresponds to such *recA* mutant cells without chromosomes [28]. We propose that DNA degradation by RecBCD extends further around DSBs, degrading the entire chromosome in the 9% focus-less *recA* cells and thus preventing detection of DNA loss by MFA.

### Terminus DNA loss in the *recA* mutant is increased by the inactivation of Exo V

To test whether the lower efficiency of focus loss in the *recA* mutant results from the DNA degradation activity of RecBCD in the absence of RecA (Fig 3B), we used a *recA recB* mutant. The percentage of focus-less cells was similar in *recA recB* and *recB* mutants for the *dif* proximal site *ydeV*::*parS*_pMT1_ and for the distal sites *yoaC*::*parS*_pMT1_ and *ycdN*::*parS*_pMT1_ (Table 1, S2 Table). Furthermore, time-lapse experiments showed that focus loss occurred at the time of division, in one cell only, and was transmitted to progeny (Fig 4A right panel). The frequency of initial events (21%, Table 1, Fig 4A right panel) and the high rate of transmission to progeny (83.7%) were similar in *recA recB* to the RecA^+^ *recB* strain. Furthermore, the MFA profiles were similar in *recA recB* and *recB* mutants (Fig 1C, Fig 4B right panel, S1 and S2 Figs). This result shows that in a *recA* single mutant the frequency of terminus DNA loss is reduced due to the presence of RecBCD.

In a *recA recD* mutant, DSBs are not repaired because homologous recombination is inactivated by the *recA* mutation, and dsDNA ends are slowly degraded because the *recD* mutation inactivates the Exo V activity of the RecBCD complex (the RecB nuclease is not active in the RecBC complex lacking RecD, reviewed in [20–22]). *recA recD* mutant chromosomes were analysed by MFA (Fig 4B middle panel, S2 Fig). Terminus chromosome degradation covered a much larger region and was less steep than in *recB* cells, but was still centred on *dif*, the region of GC skew inversion. We propose that terminus DSBs occur in *recA recD* cells and that the very broad zone of DNA degradation around the terminus is due to the processive and potent helicase activity of RecBC, which in the absence of RecD produces ssDNA from dsDNA ends efficiently, and thus facilitates the action of ssDNA exonucleases [23,29]. Microscopy experiments confirmed DNA loss of a larger terminus region in the *recA recD* compared to *recB* mutant cells, since 27.3% of them lacked the *dif*-proximal *ydeV-parS*_pMT1_ focus, 23% lacked the *dif*-distal *yoaC-parS*_pMT1_ focus and only 11% lacked the *ycdN*::*parS*_pMT1_ locus, the furthest from *dif* (Table 1, S2 Table). Time-lapse microscopy analysis of *ydeV-parS*_pMT1_ foci in *recA recD* cells showed that focus loss occurred as in the *recB* mutant: most often at the septum, always at the time of cell division and in one daughter cell only, and it was transmitted to the progeny (Fig 4A middle panel, another example is shown in S4 Video). The frequency of initial events was 16.1% and these events were transmitted to progeny in 65% of the cases, without cell elongation (Table 1, Fig 4A middle panel). We conclude that terminus DNA loss is limited in *recA* cells by the Exo V activity of RecBCD.

**Fig 5:**
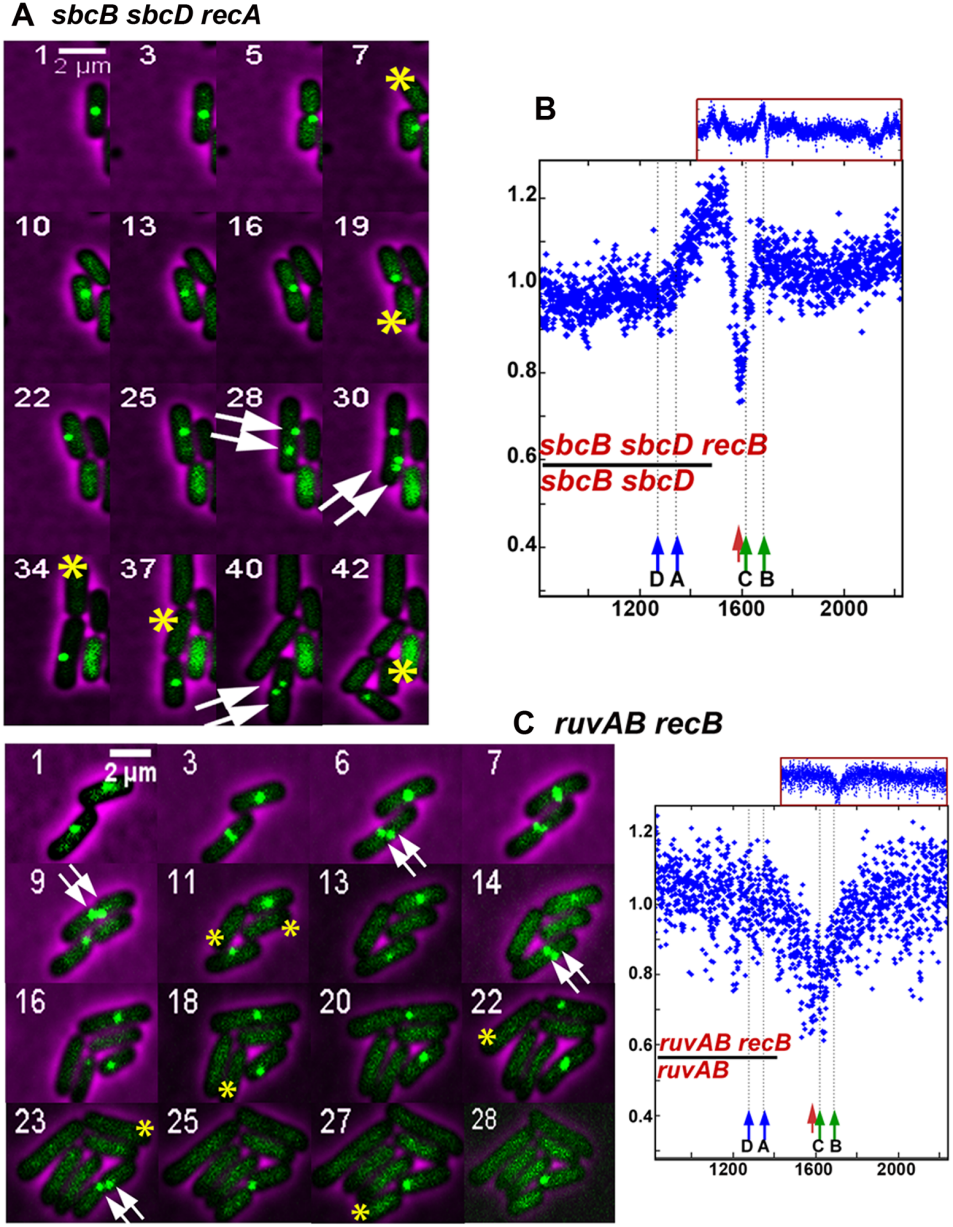
Terminus DNA loss in *recA sbcB sbcD*, *recB sbcB sbcD* and in *recB ruvAB* mutants. A and C left panel: time-lapse experiments. Examples of heritable focus loss are shown in *recA sbcB sbcD* and in *ruvAB recB* mutants. Time-lapse experiments were carried out as in Fig 4. The numbers in the upper left corner of the pictures indicate the frame numbers. The double white arrows indicate the presence of two foci before division, which shows that focus loss results from the degradation of a DNA sequence that has been previously replicated. The yellow stars show cells that have lost the focus following division. These focus-less cells generally do not divide while the sister cell that has kept the *ydeV*:: *parS*_pMT1_ site keeps growing and generates a focus-less cell at each division. B and C right panel MFA analysis. Ratios of DNA sequence coverage in *recB sbcB sbcD* versus *sbcB sbcD* mutants (B), and of *recB ruvAB* versus *ruvAB* mutant (C left panel) are shown. Original MFA data are shown in S3 Fig.

Recently, terminus DNA loss was also observed in a *recA sbcB sbcD* mutant [30]. In this mutant RecBCD is present but does not degrade DNA efficiently because DNA degradation requires dsDNA ends to be made blunt by SbcB and SbcCD exonucleases [31, 32]. In agreement with a lack of DNA degradation by RecBCD in the *recA sbcB sbcD* mutant, microscopy results in the *recA sbcB sbcD* mutant were similar to the *recA recB* mutant (Table 1, Fig 5A), while inactivation of only *sbcB* or *sbcCD* in the *recA* mutant had a partial effect (Table 1). Finally, our model predicts that heritable terminus DNA loss should occur at a low efficiency in a *recB sbcB sbcD* mutant, which lacks RecBCD but where DSBs are repaired by the RecFOR pathway of recombination (reviewed in Michel and Leach, 2012). Actually in this mutant initial events were decreased nearly two-fold (to around 10%, Table 1) and focus loss was less frequently transmitted to progeny (27,3% heritable events, Table 1). These results are in agreement with the repair of dsDNA ends by the RecFOR recombination pathway, even though MFA analysis suggested that *recB sbcB sbcD* mutants initiate unscheduled replication in the terminus, and an unexplained high level of focus-less cells in growing cultures suggested that additional phenomena occur in the terminus region of the *recB sbcB sbcD* mutant ([30]; Fig 5B; S3C and S3D Fig; Table 1). Altogether, these results demonstrate that both homologous recombination and RecBCD-mediated DNA degradation should be inactivated to observe heritable terminus DNA loss, as predicted from our model (Figs 2 and 3).

### Terminus DNA loss is not due to replication fork reversal

To date, only one particular replication fork breakage event is specific for *recB* and *recA recD* mutants, and those breaks result from RuvABC-catalysed resolution of a Holliday junction made by replication fork reversal [22,33]. Replication fork reversal is a reaction that involves the annealing of leading- and lagging-strand ends at a blocked fork, resulting in a dsDNA end adjacent to a Holliday junction [22,33]. In *recBC* and in *recA recD* mutants, the dsDNA end is neither recombined nor degraded, and resolution of the Holliday junction by RuvABC produces fork breakage [22,33]. Fork breakage by RuvABC in a *recB* mutant is a hallmark of replication fork reversal, and we tested a putative role of RuvABC in the production of the DSBs that lead to terminus DNA loss. The inactivation of *ruvAB* did not reduce the percentage of focus-less cells in *recB ruvAB* (37%, Table 1) or in *recA recB ruvAB* cells (38%, Table 1). Focus loss in the *recB ruvAB* and *recA recB ruvAB* mutants occurred at the time and most often at the site of cell division, in one daughter cell, and was transmitted to progeny (Fig 5C). Focus loss was quantified by time-lapse experiments in *recA recB ruvAB* cells, where only recombination-independent Holliday junctions can form. The frequency of initial events was unchanged by RuvAB inactivation (about 21%), and transmission of focus loss to progeny was slightly lower than in the Ruv^+^ *recA recB* mutant but remained high (60%). Furthermore, DNA loss in the *dif* region was still observed by MFA in the *recB ruvAB* mutant (Fig 5C, S3A and S3B Fig). We conclude that RuvAB is not required for terminus DNA loss in the *recB* mutant, which implies that replication fork reversal is not the main source of fork breakage in this mutant.

### The first focus-less cell is different from the subsequent ones

The model predicts that the focus-less cell generated by the first cell division carries a truncated linear chromosome lacking all sequences between the original random DSB and the terminus, therefore potentially lacks essential genes. In contrast, focus-less cells generated in the following generations, which are delimited by two DSB events in the terminus region, contain a complete linear chromosome. This prediction could be tested by comparing the ability to propagate of these two types of focus-less cells. For this experiment we had to use a *hipA hipB* deleted strain since this toxin-antitoxin locus is adjacent to *dif* and its degradation in *ydeV-parS*_pMT1_ focus-less cells prevents proliferation [34,19,35]. In a *hipA recB* mutant 30% of the first focus-less cells did not divide while all the second focus-less cells divided (<3% did not divide, Table 2). This indicates that 30% of the first focus-less cells lacked some essential proteins that were expressed by the second focus-less cells. This is in agreement with the proposal that the first focus-less cells originally carry a truncated linear chromosome and thus differ from the subsequent focus-less cells that are born with a full linear chromosome.

**Table 2:** Number of divisions made by the first and the second foci-less cells.

	Number of cells that make 0, 1 or 2 divisions			Total cells analysed
	0 division	1 division	2 divisions	
First focus-less cell	15 (30%)	30 (60%)	5 (10%)	50
Second focus-less cell	0 (<3%)	27 (87%)	4 (13%)	31

### Formation of a focus-less cell is not heritable in cells with a linear chromosome

**Fig 6:**
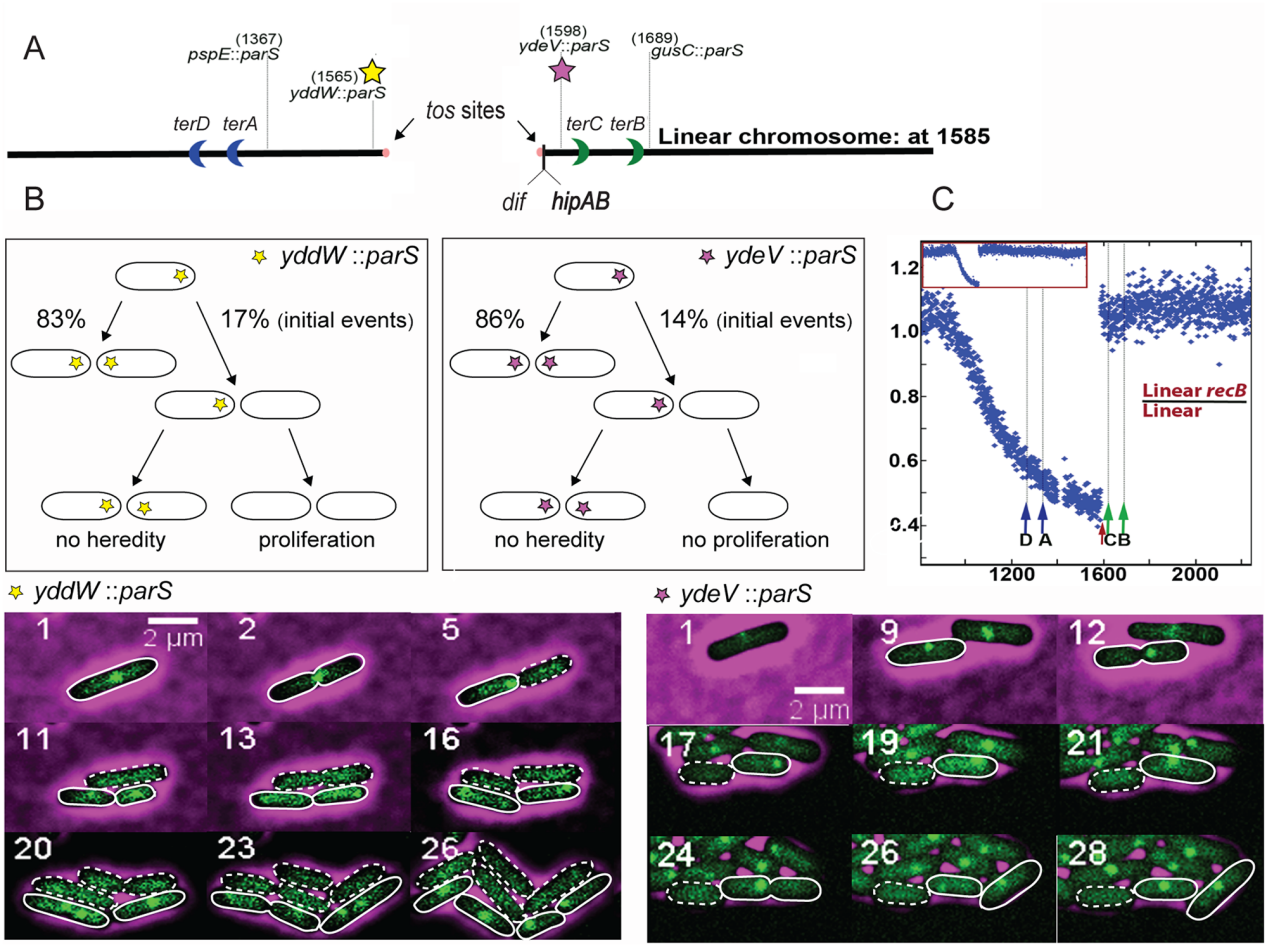
A Focus-less cell can form from any of the two ends of a chromosome linearized 3 kb from *dif*. (A) Schematic representation of the terminus region in a linear chromosome interrupted at position 1585. *terD* to *terB*, *dif*, the *parS* sites used for microscopy experiments, and the *hipA hipB* genes are shown. (B) Schematic representation of non-heritable focus loss on linear chromosomes and micrographs showing examples of focus loss during growth of *recB* cells in which the chromosome is linearized 3 kb from *dif* and carries either *yddW*:: *parS*_pMT1_ (left panels) or *ydeV*:: *parS*_pM T1_ (right panels). Time-lapse experiments were carried out as in Fig 4A. Cells that generate a focus-less cell during division are circled with a full white line. Cells that have lost the focus are circled with a dashed white line. Loss of the *yddW*::*parS*_pMT1_ focus occurring in image 5 (left panel) is not heritable, but focus-less cells divide because the *hipA hipB* genes are intact. Loss of the *ydeV*::*parS*_pMT1_ focus occurring in image 17 (right panel) is not heritable, and focus-less cells do not divide because *hipB* is degraded. Another example of *ydeV*:: *parS*_pMT1_ focus loss from a linear chromosomes is shown in S6 Video and the complete movie corresponding to the *yddW*:: *parS*_pMT1_ images shown here is shown in S7 Video. C. Ratio of normalized sequence reads in RecB+ over *recB* mutant cells with a linear chromosome. Because *hipAB* is next to *dif*, cells that degrade this chromosome end do not multiply because they are blocked by the HipA toxin and become underrepresented in the population. Cells that degrade the other chromosome end multiply, which increases their relative amount in the population. Consequently, DNA loss in the population is amplified on the *yddW*::*parS*_pMT1_ side and underestimated at the other end. Original normalized profiles used to calculate ratios are shown in S4 Fig. We observed that our linear strain carries a deletion of about 50 kb around positions 1400 to 1450, which was not observed previously and may be specific for our isolate.

According to the model presented in Fig 2, transmission of the phenomenon to progeny requires the production of a σ-replicating chromosome, in which a linear and a circular chromosome are attached by a replication fork in their terminus (Fig 2 Step E). Therefore transmission should be prevented by using cells in which the naturally circular *E*. *coli* chromosome has been converted to a linear chromosome, artificially interrupted in the *dif* region. We used a strain that carries the terminus sequence *tos* of the linear phage N15, 3 kb from *dif* on the right replichore, and that expresses the N15 telomerase TelN, which processes the *tos* sequence (Fig 6A) [36]. This strain propagates with a linear chromosome, interrupted 3 kb from *dif* [36]. As a control for these experiments, we used an isogenic strain with a circular chromosome, which carries the *tos* site but lacks the gene encoding the TelN protein (S1 Table).

Cells with linear chromosomes were studied by fluorescence microscopy, using *ydeV*::parS_pMT1_ or *gusC*::parS_pMT1_ markers on the left replichore (13 kb or 105 kb from the chromosome end, respectively), and *yddW*::*parS*_pMT1_ or *pspE*:: *parS*_pMT1_ markers on the right replichore, (19 kb or 217 kb from the chromosome end, respectively) (Fig 6A, Table 3). It should be noted that the *hipA hipB* locus is adjacent to *dif*, therefore it will be degraded together with the *ydeV*::parS_pMT1_ or *gusC*::parS_pMT1_ markers, inhibiting growth of these focus-less cells. In contrast, because it is separated from the other chromosome arm by the *tos* site, it will remain intact in cells that lose the *yddW*::*parS*_pMT1_ or *pspE*:: *parS*_pMT1_ markers, allowing the multiplication of the cells that lose these loci.

**Table 3:** Terminus DNA loss in the linear chromosome.

genotype	% cells with 0 focus			
	*pspE*::*parS*_pMT1_	*yddW*:: *parS*_pMT1_	*ydeV*::*parS*_pMT1_	*gusC*::*parS*_pMT1_
Circular *tos*	1.4 ± 0.3	1.4 ± 0.7	1.8 ± 0.7	ND
Circular *tos recB*	15.3 ± 3.2	29.6 ± 2.6	31.9 ± 2	ND
Linear *tos*	1.1 ± 0.01	4.1 ± 2.8	4.8 ± 0.14	2.4 ± 0.02
Linear *tos recB*	56.4 ± 1.6	59.6 ± 6.2	20.7 ± 1.4	10.5 ± 0.9

The linear chromosome ends are shown in Fig 6A. Positions of the important locus are, from left to right: *pspE*::*parS*_pMT1_ (1367 kb), *yddW*:: *parS*_pMT1_ (1565 kb), *tos* linearization site (1585kb), *dif* site and *hipA hipB* operon (1588 kb), *ydeV*::*parS*_pMT1_ (1598kb), *gusC*::*parS*_pMT1_ (1689 kb). Numbers indicate nucleotide coordinates.

The proportion of cells lacking the end-proximal *ydeV*::parS_pMT1_ focus increased from 4.8% in the RecB^+^ strain to 20.7% in the *recB* mutant, while the proportion of cells lacking the end-distal *gusC*::parS_pMT1_ focus reached 10.5% in the *recB* mutant (Table 3). In contrast, the proportion of the cells devoid of the end-proximal *yddW*::parS_pMT1_ focus increased from 4.1% in RecB^+^ to nearly 60% in the *recB* mutant, while the proportion of cells lacking the end-distal *pspE*::parS_pMT1_ focus reached 56% in the *recB* mutant. As expected, in control isogenic strains with a circular chromosome, the proportion of cells lacking the *dif*-proximal loci (*ydeV*::parS_pMT1_ or *yddW*::parS_pMT1_) was increased from about 1% in RecB^+^ to around 30% in the *recB* mutant, and was higher than the loss of a *dif*-distal locus (*pspE*::parS_pMT1_, 15% focus-less cells in a *recB* mutant, Table 3). The difference between right and left replichores was specific for linear chromosomes, suggesting that the proportion of focus-less cells could be largely influenced by the position of the *hipA hipB* locus. To precisely quantify terminus DNA loss, *ydeV*::parS_pMT1_ and *yddW*::*parS*_pMT1_ foci were analysed in *recB* by time-lapse microscopy experiments.

Results in the control *recB* mutant that carries *tos* but harbours a circular chromosome owing to the absence of TelN protein were similar to those observed in MG1655, with a loss of *ydeV*::parS_pMT1_ or *yddW*::*parS*_pMT1_ foci occurring at the time of cell division, in one of the two daughter cells, and transmitted to progeny (S5 Video). We counted 15.9% initial events for the *yddW*::*parS*_pMT1_ locus and more than 80% of the events were transmitted to progeny (Table 4). In cells with a linear chromosome, a similar percentage of initial events was observed with the terminus-proximal markers on the left and right replichores (14–17%) but, importantly, the phenomenon was generally not transmitted to progeny, as only 11 to 19% of the events were heritable (Table 4, note that this level corresponds to the percentage of initial events and could therefore correspond to independent events occurring by chance after a first one). This result indicates that the transmission of focus loss to the progeny requires circularity of the chromosome.

**Table 4:** Loss of focus is not transmitted to progeny in cells harbouring a linear chromosome.

strain	Initial Events	Transmitted
*ydeV*:: *parS*_pMT1_ circular *recB* (a)	17.7% (350)	74.5%
*ydeV*:: *parS*_pMT1_ linear *recB*	14% (785)	18.9%
*yddW*:: *parS*_pMT1_ circular *recB*	15.9% (521)	85.2%
*yddW*:: *parS*_pMT1_ linear *recB*	17.5% (405)	10.8%

Results are the sum of two independent experiments.

Because MG1655 *tos* isogenic to the linear strain behaves as MG1655 in snapshot experiments (Table 3), these results are from our MG1655 (Table 1).

In addition, time-lapse experiments allowed us to observe that *ydeV*::parS_pMT1_ focus-less cells did not multiply, as expected from the concomitant degradation of the *hipA hipB* locus (Fig 6B right panels, another example of *ydeV*::parS_pMT1_ focus loss from a linear chromosome is shown in S6 Video). In contrast, cells that lose the *yddW*::*parS*_pMT1_ locus on the right replichore could multiply for at least three generations (Fig 6B left panels, complete movie is shown in S7 Video). Therefore, the high level of *yddW*::parS_pMT1_ and *pspE*::*parS*_pMT1_ focus-less cells can be simply explained by the propagation of focus-less cells carrying an intact *hipA hipB* locus. Genomes of the RecB^+^ and *recB* mutant linear strains were analysed by MFA (Fig 6C, S4 Fig). A depletion of DNA sequences was observed on one chromosome arm, while nearly no DNA loss was observed on the chromosome arm carrying *hipA hipB*, possibly because the MFA technique is not sensitive enough to detect the weak level of *recB*-dependent DNA loss on this arm (16%, Table 3). Although the MFA profile was therefore not informative regarding terminus DNA loss, it was in full agreement with the microscopy results.

We conclude from these experiments that focus-less cells, which reflect a lack of terminus DNA, could be observed at either of the two ends of a *recB* mutant chromosome linearized at position 1585 kb. The phenomenon shares some common features with terminus DNA loss observed in circular chromosomes (focus loss in one daughter cell, at the time of division), but, importantly, the capacity to lose terminus DNA in one daughter cell was not heritable. These results indicate that chromosome circularity, and thus DNA continuity of the terminus region is required for the heredity of the phenomenon, although it is not required for the formation of a first focus-less cell (initial events).

### Inheritance in the *recA* mutant depends on Tus

According to our model, transmission of terminus DNA loss to progeny depends on the persistence of the short DNA tail formed at each generation by septum closure until the arrival of the following replication round (Fig 2 step D). In a *recB* mutant, DNA degradation is mediated by the action of helicases and exonucleases and is expected to be much slower than RecBCD-catalysed DNA degradation [37,38]. In a *recA* mutant, this short tail is the target of the potent RecBCD Exo V activity and should be efficiently degraded, which explains why only 37% of the initial events, instead of 80% in the *recA recB* mutant, were transmitted to progeny at least for one generation. The length of this tail is defined by the distance between the site of breakage (the *dif* region) and the position at the time of division of the intact replication fork that is slowed down by *ter* sites (Fig 2 step C). Therefore, the duration of replication blockage at *ter* is expected to control heredity of terminus DNA loss in a *recA* mutant. We measured terminus DNA loss in a *tus recA* mutant, in which replication forks do not arrest at *ter*. *tus* inactivation increased the percentage of initial events from 7% to 11%, and increased the percentage of heritable events in the *recA* mutant from 37% to 64%, similar to the *recA recD* level (Table 1, S2 Table). This result shows that in a *recA* mutant replication arrest at *ter* limits terminus DNA loss and particularly the transmission of terminus DNA loss to the progeny.

### MatP inactivation reveals a post-replicative attachment of the two terminus loci in the *recB* mutant

**Fig 7:**
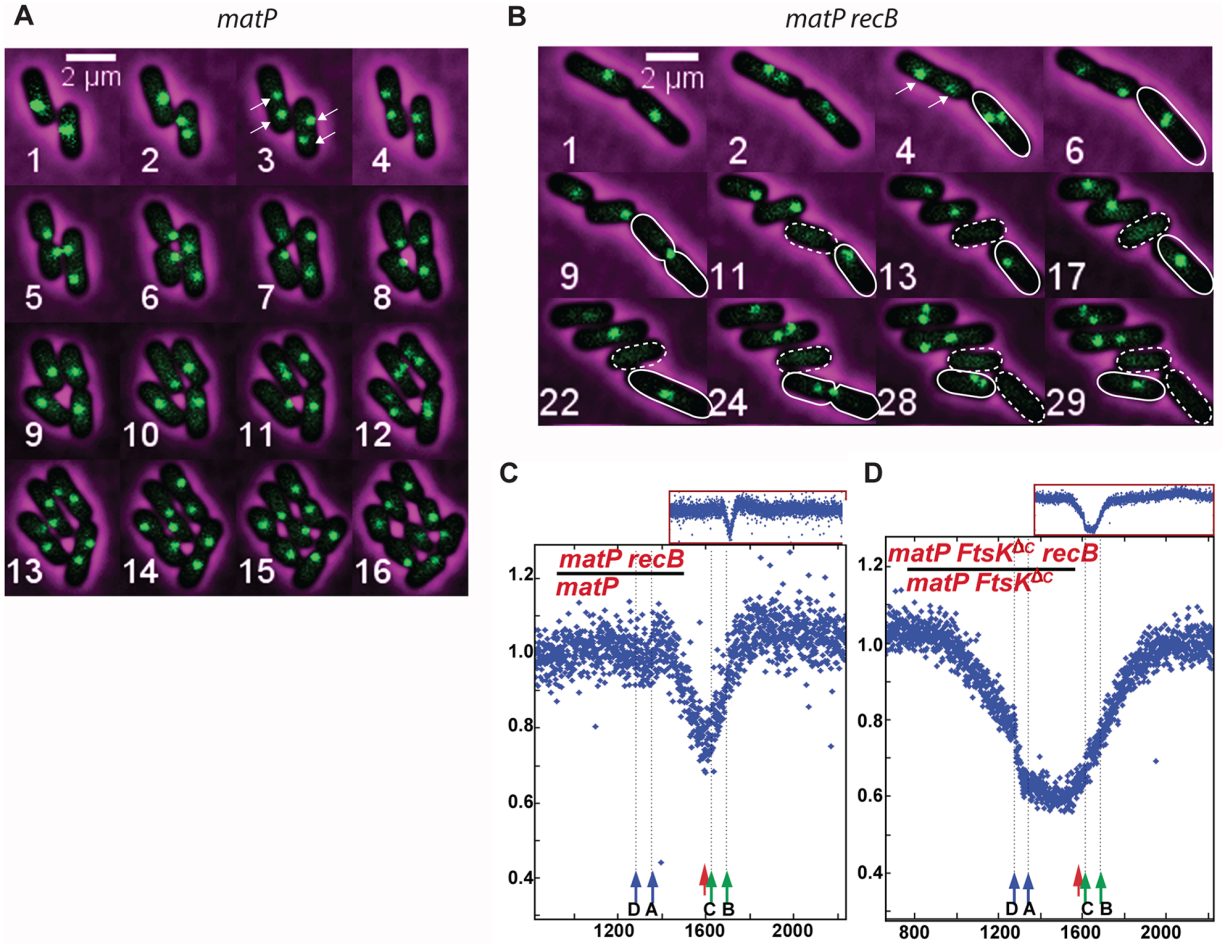
Terminus DNA loss occurs in a *matP recB* mutant. (A) and (B) Micrographs showing *ydeV*:: *parS*_pMT1_ focus behaviour during growth of *matP* and *matP recB* cells. Arrows indicate segregating *ydeV*:: *parS*_pMT1_ foci. Cells that generate a focus-less cell during division are circled with a full white line. They contain non-segregating *ydeV*:: *parS*_pMT1_ foci and give rise to a focus-less cell (circled with a dashed white line) in a heritable way (image 13 and 28). (C) Ratios of normalized reads in isogenic *matP recB* mutants and *matP* RecB^+^, (D) Ratios of normalized reads in isogenic *matP ftsK*^*ΔCTer*^ *recB* mutants and *matP ftsK*^*ΔCTer*^ RecB^+^ cells. Ratios are plotted against chromosomal coordinates (in kb) and original normalized profiles used to calculate ratios are shown in S5 Fig.

The model presented in Fig 2 implies that the two terminus sequences remain covalently attached. In wild-type cells, this covalent attachment cannot be directly visualized, as the two newly-synthesized terminus regions are anyway co-localized at the septum position when MatP is present. In contrast to wild-type cells, in a *matP* mutant terminus sequences readily separate after replication [6,7]. We used a *matP* mutant to test the attachment of the newly synthesized terminus sequences in the *recB* mutant. As previously described, all *matP* cells exhibited an early segregation of the *ydeV*::*parS*_pMT1_ loci to the ¼ and ¾ positions in the cell, owing to the lack of attachment of the terminus macrodomain to the septum ([6] arrows in Fig 7A).

MFA and microscopy experiments showed that terminus DNA loss occurred in *matP recB* as in the *recB* single mutant (Table 1, Fig 7B and 7C, S6 Fig). In time-lapse experiments, focus loss occurred at the septum, at the time of division, in one of the two daughter cells, and in a heritable manner (Fig 7B). Measures of initial events and heredity showed that DNA loss was unaffected by *matP* inactivation (15.5% initial events, 86.4% heredity; Table 1). However, although in most *recB matP* cells *ydeV-parS*_pMT1_ foci segregated prematurely to the ¼ and ¾ positions (arrows in Fig 7B), in ~15–16% of cells foci remained together at the site of septum formation until division (cells circled with a full white line in Fig 7B). Interestingly, focus loss occurred specifically in those cells where the two replicated *ydeV*::*parS*_pMT1_ foci remained nearby in the division plane, or, in other words, the lost focus was always one of the two foci that remained at the septum position after replication, in spite of the absence of MatP (Fig 7B, focus-less cells are circled with a dashed white line). The specific loss of one of the two non-segregated loci in the *matP recB* mutant supports the idea that the two replicated chromosomes are linked at a position close to the *ydeV* locus (Fig 2).

FtsK also contributes to the positioning of the chromosome terminus at the septum via binding of its C-terminal domain to KOPS sequences and chromosome translocation [5,39]. Nevertheless, in the *matP ftsK*^ΔCter^ *recB* mutant, which lacks the two functions known to position the terminus at the septum, ~40% focus-less cells were observed (Table 1). The MFA experiment showed an enlarged degraded region confirming that FtsK is not required for terminus DNA loss. Furthermore, DNA degradation was no longer centred on *dif* and spanned the entire fork trap, delimited by oppositely-oriented *ter* sites (Fig 7D), confirming that FtsK translocation activity is responsible for the localization of the peak of DNA degradation around *dif*. Importantly, terminus DNA loss is observed in the absence of the functions that position the chromosome terminus at the septum, which supports the idea that the two terminus sequences are attached covalently.

### Mutant cells that undergo terminus DNA loss show a *dif*-specific segregation defect

**Fig 8:**
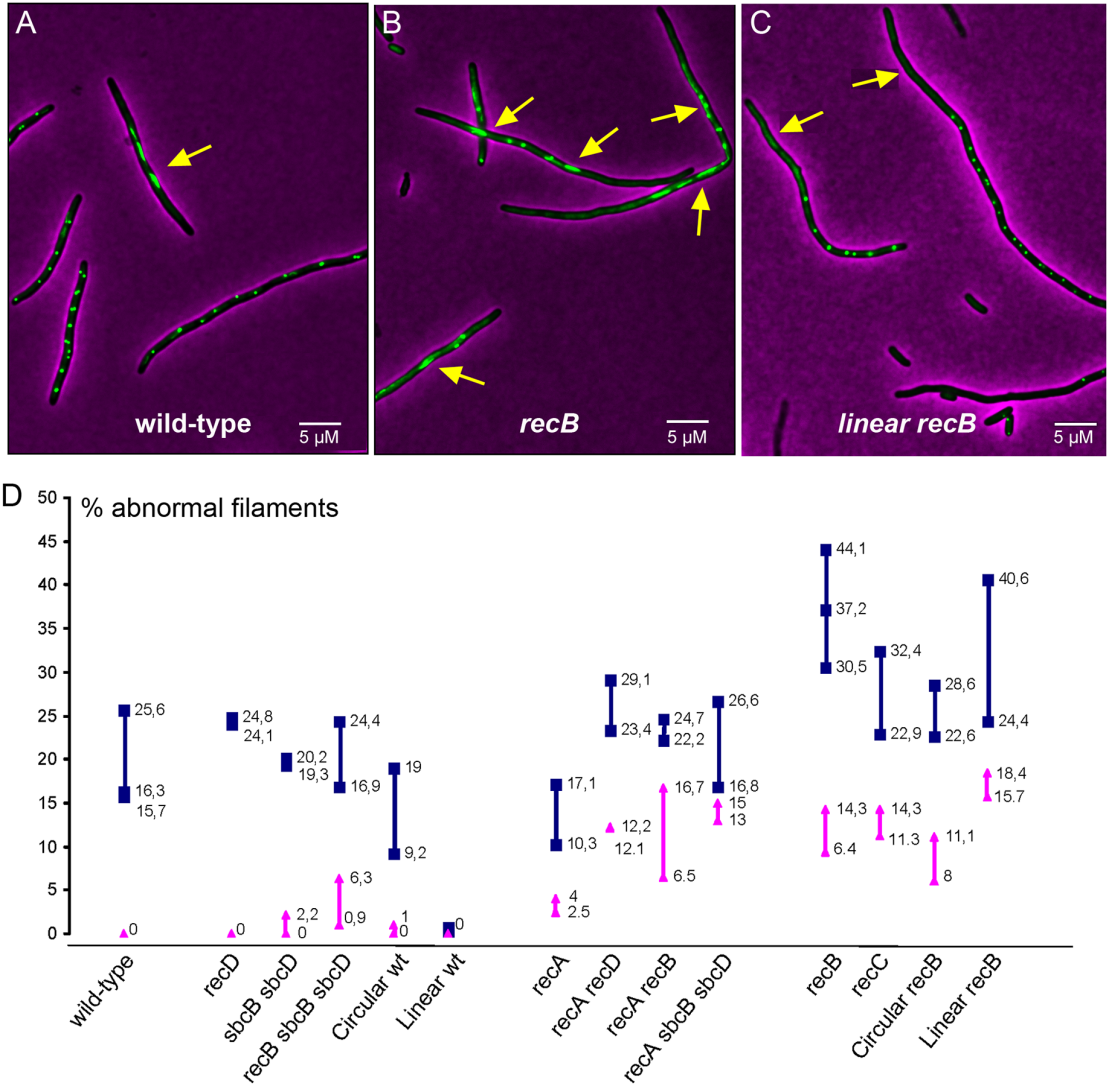
Cephalexin treatment reveals a lack of terminus segregation. A, B and C Micrographs show examples of highly intense, non-segregating *ydeV*:: *parS*_pMT1_ foci (A and B, yellow arrows) or regions in the filaments that are devoid of *ydeV*:: *parS*_pMT1_ focus (C, yellow arrows). D Percentage of cells with abnormal filaments in various mutants. In all strains except for the linear *recB* mutant, abnormal filaments contained focus-less regions associated with very intense, non-segregated foci (as shown in panel A and B). In the linear *recB* mutant, non-segregated highly intense foci were not observed, and abnormal filaments showed focus-less regions associated with well-segregated foci (as shown in panel C). Because of some variations from experiment to experiment, all results are shown, and we cite the two extreme values in the text. Blue square and blue line *ydeV*:: *parS*_pMT1_ foci, pink triangles and pink lines *yoaC*:: *parS*_pMT1_ foci.

To confirm the post-replication attachment of two terminus regions in a MatP^+^ strain, we analysed chromosome segregation using cells where division is blocked by cephalexin, an inhibitor of the late septum protein FtsI [40]. As expected, cephalexin treatment caused the formation of elongated cells, and most wild-type cells showed regularly spaced *ydeV*::*parS*_pMT1_ foci, while 15–25% showed non-segregated foci (Fig 8A and 8D). The proportion of cells with non-segregated *ydeV*::*parS*_pMT1_ foci was similar in all recombination proficient cells: between 11% and 25% non-segregated *ydeV*::*parS*_pMT1_ loci (*dif* proximal) and between <0.5% and 6.3% non-segregated *yoaC*::*parS*_pMT1_ loci (300 kb away from *dif*) (Fig 8D, see wild-type, *recD*, *sbcB sbcD*, *recB sbcB sbcD* and the circular chromosome control cell). Septum assembly is essential for dimer resolution owing to the role of the FtsK C-terminal domain in XerCD activation [41,42], and about 15% of cells contain a chromosome dimer [43]. Consequently, the percentage of recombination proficient cells showing non-segregated *dif*-proximal loci can be accounted for by the lack of dimer resolution. In support of this idea, because dimers only form in circular chromosomes, nearly all cells harbouring a linear chromosome showed proper segregation of *ydeV*::*parS*_pMT1_ loci upon cephalexin treatment (0.7% non-segregated, Fig 8D).

In a *recA* mutant, 10–17% cells showed non-segregated *ydeV*::*parS*_pMT1_ foci. Since chromosome dimers do not form in the absence of homologous recombination (*recA* mutant), these 10–17% cells suffer a dimer-independent segregation defect (Fig 8D). The marker further from *dif* (*yoaC*::*parS*_pMT1_) was less affected and showed only 4% non-segregated cells. Inactivation of *recD*, *recB* or *sbcB sbcD* in the *recA* mutant increased the proportion of cells showing non-segregated *ydeV*::*parS*_pMT1_ foci to 17–29%, therefore, in *recA* mutants the lack of segregation of the *ydeV*::*parS*_pMT1_ foci after cephalexin treatment (Fig 8D) is correlated with the frequency of initial loss events (Table 1). This result supports the idea that terminus DNA loss occurs in cells in which the two termini remain covalently linked after replication.

Interestingly, in the *recB* and *recC* mutants the percentage of cells presenting a segregation defect was as high as 22–44% for the *dif* proximal locus and increased to 9–14% for the *dif*-distal locus (Fig 8B and 8D). Since dimer formation is half as frequent in the *recB* mutant as in wild-type cells [43], the proportion of cells in which *ydeV*::*parS*_pMT1_ foci did not segregate independently of dimer formation could be as high as 15–30%, as in *recA recD* and *recA sbcB sbcD* mutants. This percentage correlates with the level of terminus DNA loss observed in dividing cells (nearly 20% of initial events). Note that in cephalexin-treated cells focus segregation was similar to wild-type in *recB sbcB sbcD* (Fig 8D), although this mutant showed an intermediate level of initial events between wild-type and *recB* mutant, (10%, Table 1). To account for this observation, we propose that dsDNA end repair is slower when catalysed by RecFOR and RecA (*recB sbcB sbcD* cells) than when catalysed by RecBCD and RecA (wild-type). Consequently, in *recB sbcB sbcD* cells that do not divide (cephalexin treated), initial DSBs are repaired, although slowly, which allows segregation of sister chromosomes, while in dividing cells σ-replicating chromosomes are not always repaired prior to division and are sometimes cleaved. Finally, as expected from its high level of initial events, the *recB* mutant with a linear chromosome showed a high level of cells with an abnormal pattern of *ydeV*::*parS*_pMT1_ foci after cephalexin treatment (24–40%). However, in the linear chromosome *recB* mutant the abnormal cephalexin-induced filaments presented a deficit of *ydeV*::*parS*_pMT1_ foci (Fig 8C) instead of non-segregated foci, as observed in *recB* cells and in other mutants with a circular chromosome (Fig 8B). As described below this is expected from the random breakage of one replication fork in a linear chromosome (S6 Fig, see Discussion).

In conclusion, a defect in segregation of the two replicated *dif* regions is observed in cells that lack homologous recombination and Exo V mediated DNA degradation both in the presence (in a *matP* mutant) and the absence (in cephalexin-treated cells) of cell division. This finding supports the idea that terminus DNA loss results from septum closure on non-separated chromosome termini.

## Discussion

We propose here that the terminus DNA loss observed in a *recB* mutant results from septum-induced breakage in the terminus of σ-replicating chromosomes, and transmission of the σ-replicating structure to progeny (Fig 2). As predicted from this model, we show here that the phenomenon of terminus DNA loss observed in *recB* mutant cells at the time of division (1) only occurs when homologous recombination is inactivated and dsDNA end degradation is limited (Fig 4, Table 1), (2) generates a first focus-less cell that differs from the following one by being less capable of cell division (Table 2), (3) is not transmitted to progeny when the chromosome is interrupted in the terminus (Fig 6, Tables 3 and 4), (4) is more efficiently transmitted to progeny in a *recA tus* than in a *recA* mutant (Table 1), and (5) is associated with segregation defects of the two sister terminus sequences (Figs 7B and 8). The model also predicts that this class of terminus DSBs does not occur in wild-type cells where the original random DSB can be repaired by RecBCD and RecA. Accordingly, by measuring RecA binding in wild-type cells, we could not detect an increased occurrence of DSB repair in the terminus region compared to the rest of the chromosome, in conditions where RecA binding to a known DSB was readily detected [19].

### Terminus DNA loss in a *recA* mutant

Initial events rely on the persistence of a σ-replicating chromosome tail after fork breakage, which can lead to a focus-less cell only if the linear tail is neither degraded nor recombined, and segregates to the future daughter cell (Fig 2B and 2C). The observation that initial events are three-fold less frequent in *recA* than in a *recA recB* mutant suggests that in two thirds of cases the potent Exo V activity of RecBCD (variable but up to 800–900 bp per sec, [44,45]) catches up with the progressing fork (500–600 bp per sec, [46,47]) and fully degrades this first long tail, which prevents initial events (Fig 3B pathway B). In a *recA* mutant the frequency of both initial and secondary events is increased by *tus* inactivation. The increase of initial events could be explained by two ways. Firstly, complete DNA degradation of the first tail is expected to be delayed by the progression of the active replication fork across the terminus. Secondly, in a subpopulation of cells, the progression of one of the two intact replication forks beyond the terminus, in the direction opposite to the main transcription direction, might increase replication fork blockage, as previously proposed, and in turn replication fork breakage and σ-replicating chromosome formation [48,49]. Increased heredity in the *recA tus* compared to the *recA* mutant supports the idea that heredity relies on the persistence of the truncated tail after terminus DNA breakage, hence on the length of this tail (Fig 2D–2F).

Growing cultures of *recA* mutants were reported to contain 5 to 10% anucleate cells (see for example [32,50]), which corresponds to the percentage of focus-less cells observed in this work. Interestingly, in the *recA* mutant we did not observed loss of *parS*_pMT1_ foci at any time other than cell division. This observation suggests that most anucleate cells in MM cultures of a *recA* mutant result from the degradation of a linear chromosome formed by two successive DSBs: one at a random position during replication and one close to *dif* during septum closure (Fig 3B pathway C).

### Terminus DNA loss in a linear chromosome

The formation of a focus-less cell is not transmitted to progeny when the chromosome is linearized by *tos*/TelN, in agreement with the idea that heredity requires circularity of the chromosome for the merging of the intact replication fork with the following replication round (Fig 2). A model showing the events expected to occur in the *recB* mutant harbouring a linear chromosome, according to the model shown in Fig 2, is presented in S6 Fig. In the *recB* mutant with a linear chromosome, accidental breakage of one replication fork, while the other replication fork progresses to the chromosome end, leads to a linear head-to-head dimer composed of one entire chromosome and one truncated chromosome, linked by the telomerase TelN recognition site (S6 Fig, 3 first steps). The two halves of this dimer segregate to the two future daughter cells, with the TelN recognition site at mid-cell. TelN action at this site produces an intact linear chromosome, which segregates to form the focus-carrying cell, and a truncated chromosome (focus-less cell). Cells that harbour a truncated chromosome lacking the *ydeV* site do not multiply while those that lack the *yddW* locus multiply. Note that the reaction starts by fork breakage as on a circular chromosome, but the missing terminus, which fails to be copied by the broken replication fork, is not copied by the other fork (and then degraded), since the chromosome is linear (S6 Fig, progression of the intact fork to the end). Accordingly, in time-lapse experiments we did not observe a duplication of the *ydeV*::parS_pMT1_ or *yddW*::*parS*_pMT1_ focus prior to focus loss (Fig 6), and after cephalexin treatment abnormal elongated cells showed regions devoid of focus (Fig 8). Linearization in the terminus by TelN separates the intact from the truncated linear chromosomes after replication completion (S6 Fig, last step), and no DSB occurs during cell division.

### Is the formation of σ-replicating chromosomes responsible for the low viability of a recBC mutant?

Our results account for the long-standing observation of three types of cells in a *recB* mutant culture: non-dividing cells (our focus-less cells), residually dividing cells (the cells that produce a focus-less cell), and normally dividing cells [51], Furthermore, the viability of *recB* cells is lower than that of *recA* mutant cells although, in addition to DSB repair, the latter also lack single-strand gap recombinational repair and induction of all DNA repair genes under the control of the SOS response [51–53]. It was proposed that the tail of a σ-replicating chromosome is a lethal form of damage in a *recBC* mutant, and that σ-replicating chromosomes are less deleterious in a *recA* mutant where the linear tail can be degraded by RecBCD [26,27,52]. Our study strongly supports the idea that σ-replicating chromosomes are the major cause of the low viability of the *recB* mutant but they do not simply cause lethality. Instead, one cell remains alive while most of the tail is segregated and cleaved off into a doomed daughter cell at each generation.

Several kinds of replication impairments render RecBC, and sometimes also RecA, essential for viability [54,55]. The reverse assumption, that the viability defect of *recBC* and *recA* mutants directly reflects a correspondingly high level of spontaneous replication impairment, was often postulated. However, in contrast with this assumption, flow cytometry and MFA analyses showed that chromosome replication proceeds with a rate similar to wild-type in *recB* and *recA* mutants [19,56]. Replication fork blockage or breakage was not observed, although it should have been detected if it were responsible for the low viability of these mutants. Our model provides an explanation for this paradox. Our data allow us to determine for the first time that the level of spontaneous replication fork breakage is ~18% per cell per generation (9% per fork), which is too low to be directly detected in population studies. Finally, our findings raise future questions to be addressed: how does spontaneous replication fork breakage occur, and how are terminus DSBs catalysed? We have previously shown that the periplasmic endonuclease Endo 1 is not involved [19] and no nuclease has been reported to be specifically associated with the septum.

## Materials and methods

### Strains

Strains are described in S1 Table. Most strains were constructed by P1 transduction. New mutations were constructed as described in [57], using DY330 [58]. Oligonucleotides used for constructions and mutation checking are shown in S3 Table. *recA* and *recB* mutations were checked by measuring UV sensitivity. *recD* mutations were checked by comparing the plating efficiencies of wild-type T4 and T4*gpIIam* phages (the unprotected T4*gpIIam* only multiplies on *recBC* and *recD* mutants [59]). *sbcCD* mutations were checked by comparing the plating efficiencies of wild-type λ a λ carrying a long palindrome (the λDRL154 phage that carries a long palindrome only multiplies on *sbcCD* mutants, [60]). In the course of this work, we fortuitously discovered that our microscopy strains are Phi80 lysogens. In contrast with the reported effects of Phi80 lysogeny in AB1157 [61,62], Phi80 lysogens in MG1655 are only very weakly UV sensitive (around 10% survival at 40 J/m2), do not affect T4 or λ phages plating, and do not show a *recD* or *sbcCD* mutant phenotype. These background differences presumably result from the high divergence of the AB1157 and MG1655 genomes. All strains used for MFA are Phi80-free and experiments with Phi80-free *recA* and *recB* mutants confirmed that the cryptic phage did not affect the microscopy results (S4 Table).

### Marker frequency analysis

MFA were performed and analysed as described in [19], with cells grown in M9 glucose at 37°C. The MFA data have been submitted to the ArrayExpress repository. The access number for these data is E-MTAB-6122.

### Microscopy analyses

Microscopy experiments were performed and analysed as described in [19]. For snapshot analysis cells were grown in M9 glucose at 37°C. Time-lapse experiments were realized on M9 glucose at 30°C.

## Supporting information

S1 Table Strains used in this study. (PDF)

S2 Table Percentages of cells with zero, one or two foci in different mutants. (PDF)

S3 Table Oligonucleotides used in this study. (PDF)

S4 Table Percentage of cells with zero, one or two foci, ratio of initial events and of inherited events are independent of strain background (see Materials and methods). (PDF)

S1 Fig Marker frequency analyses. (A) wild-type. (B) *recB* mutant. Normalized replication profiles of exponentially growing cells are shown. Sequence read frequencies are normalized to the total number of reads and then the normalized reads (y-axis) are plotted against the chromosome coordinates in kb (x-axis). The approximate position of replication termination sites *terA* and *terC* and *oriC* are marked in each plot.
(PDF)

S2 Fig Marker frequency analyses. (A) wild-type, (B) *recA*, (C) *recA recB* and (D) *recA recD* mutants. See legend of S1 Fig.
(PDF)

S3 Fig Marker frequency analyses. (A) *ruvAB*, (B) *ruvAB recB*, (C) *sbcB sbcD*, and (D) *recA sbcB sbcD* mutants. See legend of S1 Fig.
(PDF)

S4 Fig Marker frequency of wild-type and *recB* mutants with a linear chromosome. See legend of S1 Fig.
(PDF)

S5 Fig Marker frequency analyses. (A) *matP*, (B) *matP recB*, (C) *matP ftsK*^*ΔCTer*^ and (D) *matP ftsK*^*ΔCTer*^ *recB* mutants. See legend of S1 Fig.
(PDF)

S6 Fig Model for the loss of terminal DNA in the *recB* mutant with a linear chromosome. In a first step, during replication progression one replication fork is accidentally broken. On the left part of the figure the left fork is broken, and on the right part of the figure the right fork is broken. The other replication fork progresses to the end of the chromosome, generating a linear dimer with an inverted duplication of the replicated right (or left) *tos* hairpin (Tel R/R (R/R), or Tel L/L (L/L) regions [63]). The replication origins segregate to the two cell halves and because the Tel R/R and Tel L/L regions are regions of KOPS convergence and MatP binding, they localize in the middle of the cell, where the septum forms. Resolution of the *tos* sites by TelN [63] creates an intact linear chromosome and a partial one that lacks all non-replicated chromosome sequences between the initial replication fork break and the terminus. The daughter cell that inherits the intact linear chromosome shows a focus and propagates normally. The one that carries the partial chromosome lacks the *yddW*::*parS*_pMT1_ or *ydeV*::*parS*_pMT1_ site, depending on the position of the initial DSB. In cells that lack *yddW*::*parS*_pMT1_ the *hipA hipB* genes are intact, and cells can multiply until they lack some essential protein. In cells that lack *ydeV*::*parS*_pMT1_ the *hipA hipB* genes are absent, and growth is prevented by the long-lived HipA protein. Blue lines, initial chromosome DNA strands; red and green lines, newly synthesized DNA strands; blue circles, replication origins; stars, *yddW*::*parS*_pMT1_ (yellow) or *ydeV*::*parS*_pMT1_ (pink) sites; dashed purple line, septum. L and R indicate the left and right *tos* hairpins, LL/ and R/R the inversely duplicated sites after replication. The position of the *dif* site is also indicated.
(PDF)

S1 Video Time-lapse microscopy of *recB* cells. Cells were mounted on an M9 glucose agarose pad and incubated at 30°C on the microscope stage. Images were captured every 10 min. The *dif/terC* region of chromosome is visualized as a green fluorescent focus by binding of GFP-ParBpMT1 protein to *ydeV*::*parS*_pMT1_. All frames are labelled. The double white arrows indicate visualization of two foci before division, the yellow stars show cells that have lost a focus following division. The focus-less cells did not divide while the cell that has kept the *ydeV*::*parS*_pMT1_ locus divided and produced a cell without foci at each subsequent generation. In this video, two heritable events are shown: the first cell on the left produced a focus-less cell in frames 7, 18, 28 and 35, and a cell on the right produced a focus–less cell in frames 28, 35, 42 and 51. Examples of rarer behaviours are also shown, as loss of two foci at division occurring (frame 44) after 4 heritable events (observed in about 10% of all heritable events), and one cell in the middle producing a focus-less cell (frame 25) and then returning to normal division. Only one focus-less cell divided in this video and such events were very rare. Other examples of *recB* mutant videos were previously published in [19].
(AVI)

S2 Video Time-lapse microscopy of *recA* cells, showing an example of heritable focus loss with a return to normal growth after two generations. Heritable focus loss rarely occurred for more than 2 or 3 generations in the *recA* mutant.
(AVI)

S3 Video Time-lapse microscopy of *recA* cells showing an example of heritable focus loss with cell elongation. The cell on the left elongates (frames 19 to 28) before producing a focus-less cell frame 31, and elongates again (frames 32 to 49) before producing a second focus-less cell frame 50. A cell on the top elongates from frame 30 to the end of the video and does not divide. Elongated cells are indicated with an “e”.
(AVI)

S4 Video Time-lapse microscopy of *recA recD* cells. Most focus loss in the *recA recD* mutant was transmitted at each generation as in the *recB* or the *recA recB* mutants, but alternative behaviours were more frequent that in *recB* and *recA recB* mutants, accounting for a slightly lower percentage of heritable events. Two examples are shown here. The cell at the top produced a focus-less cell (frames 21, 31, 39) but then returned to normal division (frame 49—this type of event was counted as heritable). The cell at the bottom produced a focus-less cell (frame 21), then underwent a normal division but each of the daughter cells produced a focus-less cell at the next generation (frame 47—this type of event was not counted as heritable).
(AVI)

S5 Video Time-lapse microscopy of *recB yddW*:: *parS*_pMT1_ cells with a circular chromosome. The cell at the top produced a focus-less cell at each division (frames 18, 39, 50) and a cell below produced focus-less cells (frame 49 and 56).
(AVI)

S6 Video Time-lapse microscopy of *recB ydeV*:: *parS*_pMT1_ cells with a linear chromosome. Focus-less cells are produced from different parental cells (frame 10, 34, and 37). After producing a focus-less cell, the focus-containing cells returned to normal growth, and focus-less cells did not divide.
(AVI)

S7 Video Time-lapse microscopy of *recB yddW*:: *parS*_pMT1_ cells with a linear chromosome. A focus-less cell was produced frame 6 and divided (frames 14, 20, 23, 29). After producing a focus-less cell, the focus-containing cell returned to normal growth.
(AVI)
