# Supplementary material for: Broken replication forks trigger heritable DNA breaks in the terminus of a circular chromosome
Source: PLoS Genet. 2018 Mar 9;14(3):e1007256. doi: 10.1371/journal.pgen.1007256 (PMC5862497; doi:10.1371/journal.pgen.1007256)
Supplement: S1 Table — (PDF) [file pgen.1007256.s001.pdf]

S1 Table Strains used in this study

Strain	Relevant genotype	Construction
JJC260	AB1157 Δ*sbcD::*Kan	Formally *orf45*::Kan, FG252 R.G. Lloyd laboratory
JJC275	Δ(*recA*-*srl*)::Tn*10* miniF-*recA*	Laboratory collection
JJC276	*recD1901*::Tn*10*	Laboratory collection
JJC671	AB1157 *ruvA60*::Tn*10* [pGB-RuvAB]	Laboratory collection
JJC777	AB1157 *recB268*::Tn*10* pDWS2	Laboratory collection
JJC1004	AB1157 Δ*sbcD::*kan Δ*sbcB::*Cm	Laboratory collection
JJC1086	AB1157 Δ*recB*::Kan	Laboratory collection Murphy et al,
JJC1176	*ftsK*$^{\Delta CTer}$ Cm	[1]
JJC1377	Δ*recB*-*recC*::Ap [pGB2-RecBCD^{+}]	[2]
JJC1392	MG1655	Laboratory collection
JJC1412	Δ*recA::*Kan [pGB2-pAra-RecA^{+}]	Δ*recA* NT 68 to 983 complemented by an arabinose-induced *recA* gene
JJC3523	Δ*lacZ* Phi80^{+}	Laboratory collection
JJC5098	DY330	[3]
JJC5507	Δ*ruvAB*::Cm	Gene replacement of *ruvAB* by FRT-Cm-FRT
JJC5650	*recB268*::Tn*10*	JJC1392 * P1 JJC777
JJC6261	*ruvA60*::Tn*10*	JJC1392 * P1 JJC671
JJC6276	*recC*::Tn*10* [pDWS2]	Laboratory collection

JJC6526	Δ*recA::*Kan	JJC1392 * P1 JJC1412
JJC6529	*ruvA60*::Tn*10* Δ*recB*::Kan	JJC6261 * P1 JJC1086
JJC6594	*recD1901*::Tn*10* Δ*recA::*Kan	JJC276 * P1 JJC1412
JJC6614	Δ*recA::*Kan [pAM-recA]	JJC6526 transformed with pAM-recA
JJC6620	Δ*sbcD::*Kan	JJC1392 * P1 JJC260
JJC6624	Δ*recA::*Kan [pAM-recA] *recB268*::Tn*10*	JJC6614 * P1 JJC777
JJC6629	Δ*recA::*Kan *recB268*::Tn*10*	JJC6624 cured of pAM-recA
JJC6646	Δ*sbcD::*Kan Δ*sbcB::*Cm *his4*	JJC6620 * P1 JJC1004
JJC6649	Δ*sbcD::*Kan Δ*sbcB::*Cm *his4 recB268*::Tn*10*	JJC6649 * P1 JJC777
JJC6932	Δ*matP::*Cm	F. Boccard laboratory collection
JJC6966	Δ*matP::*Cm	JJC1392 * P1 JJC6942
JJC6979	Δ*matP::*Cm *recB268*::Tn*10*	JJC6966 * P1 JJC777
JJC7252	Δ*lacZ ydeV* ::parS$_{pMT1}$ ::FRT *araC* ::*GFP-parB*$_{pMT1}$ ::FRT Phi80^{+}	[4]
JJC7260	Δ*lacZ ycdN* ::parS$_{pMT1}$ ::FRT *araC* ::*GFP-parB*$_{pMT1}$ ::Cm Phi80^{+}	[4]
JJC7261	Δ*lacZ ydeV* ::parS$_{pMT1}$ ::FRT *araC* ::*GFP-parB*$_{pMT1}$ ::FRT *recB268*::Tn*10* Phi80^{+}	[4]
JJC7262	Δ*lacZ ydeV* ::parS$_{pMT1}$ ::FRT *araC* ::*GFP-parB*$_{pMT1}$ ::FRT Δ*recA::*Kan Phi80^{+}	JJC7252 * P1 JJC1412
JJC7269	Δ*lacZ yoaC* ::parS$_{pMT1}$ :: FRT *araC* ::*GFP-parB*$_{pMT1}$ ::Cm *recB268*::Tn*10* Phi80^{+}	[4]
JJC7277	Δ*lacZ yoaC* ::parS$_{pMT1}$ :: FRT *araC* ::*GFP-parB*$_{pMT1}$ ::FRT	[4]
JJC7288	Δ*lacZ ydeV* ::parS$_{pMT1}$ ::FRT *araC* ::*GFP-parB*$_{pMT1}$ ::FRT Δ*ruvAB*::Cm Phi80^{+}	JJC7252 * P1 JJC5507
JJC7289	Δ*lacZ ydeV* ::parS$_{pMT1}$ ::FRT *araC* ::*GFP-parB*$_{pMT1}$ ::FRT *recD1901*::Tn*10* Phi80^{+}	JJC7252 * P1 JJC276

JJC7298	Δ*lacZ ydeV* ::parS$_{pMT1}$ ::FRT *araC* ::*GFP-parB*$_{pMT1}$ ::FRT Δ*ruvAB*::Cm *recB268*::Tn*10* Phi80^{+}	JJC7288 * P1 JJC777
JJC7305	Δ*lacZ yoaC* ::parS$_{pMT1}$ :: FRT *araC* ::*GFP-parB*$_{pMT1}$ ::FRT *recD1901*::Tn*10* Phi80^{+}	JJC7277 * P1 JJC276
JJC7309	Δ*lacZ ydeV* ::parS$_{pMT1}$ ::FRT *araC* ::*GFP-parB*$_{pMT1}$ ::FRT *recD1901*::Tn*10* Δ*recA::*Kan Phi80^{+}	JJC7289 * P1 JJC1412
JJC7310	Δ*lacZ yoaC* ::parS$_{pMT1}$ :: FRT *araC* ::*GFP-parB*$_{pMT1}$ ::FRT Δ*recA::*Kan Phi80^{+}	JJC7277 * P1 JJC1412
JJC7312	Δ*lacZ yoaC* ::parS$_{pMT1}$ :: FRT *araC* ::*GFP-parB*$_{pMT1}$ ::FRT *recD1901*::Tn*10* Δ*recA::*Kan Phi80^{+}	JJC7305 * P1 JJC1412
JJC7314	Δ*lacZ ydeV* ::parS$_{pMT1}$ ::FRT *araC* ::*GFP-parB*$_{pMT1}$ ::FRT *recB268*::Tn*10* pAM-*recBCD* Phi80^{+}	JJC7261 transformed with pAM-*recBCD*
JJC7315	Δ*lacZ yoaC* ::parS$_{pMT1}$ :: FRT *araC* ::*GFP-parB*$_{pMT1}$ ::Cm *recB268*::Tn*10* pAM-*recBCD* Phi80^{+}	JJC7269 transformed with pAM-*recBCD*
JJC7322	Δ*lacZ ydeV* ::parS$_{pMT1}$ ::FRT *araC* ::*GFP-parB*$_{pMT1}$ ::FRT *recB268*::Tn*10* pAM-*recBCD* Δ*recA::*Kan Phi80^{+}	JJC7314 * P1 JJC1412
JJC7323	Δ*lacZ yoaC* ::parS$_{pMT1}$ :: FRT *araC* ::*GFP-parB*$_{pMT1}$ ::Cm *recB268*::Tn*10* pAM-*recBCD* Δ*recA::*Kan Phi80^{+}	JJC7315 * P1 JJC1412
JJC7346	Δ*lacZ ydeV* ::parS$_{pMT1}$ ::FRT *araC* ::*GFP-parB*$_{pMT1}$ ::FRT *recB268*::Tn*10* Δ*recA::*Kan Phi80^{+}	JJC7322 cured of pAM-*recBCD*
JJC7347	Δ*lacZ yoaC* ::parS$_{pMT1}$ :: FRT *araC* ::*GFP-parB*$_{pMT1}$ ::Cm *recB268*::Tn*10* Δ*recA::*Kan Phi80^{+}	JJC7323 cured of pAM-*recBCD*
JJC7385	Δ*lacZ ydeV* ::parS$_{pMT1}$ ::FRT *araC* ::*GFP-parB*$_{pMT1}$ ::FRT *matP*::cm Phi80^{+}	JJC7252 * P1 JJC6932
JJC7389	Δ*lacZ ydeV* ::parS$_{pMT1}$ ::FRT *araC* ::*GFP-parB*$_{pMT1}$ ::FRT *matP*::cm *recB268*::Tn*10* Phi80^{+}	JJC7385 * p1 JJC777
JJC7391	Δ*lacZ ydeV* ::parS$_{pMT1}$ ::FRT *araC* ::*GFP-parB*$_{pMT1}$ ::FRT *matP*::FRT Phi80^{+}	JJC7385 excised of CmR by FRT recombination
JJC7396	Δ*lacZ ycdN* ::parS$_{pMT1}$ ::FRT *araC* ::*GFP-parB*$_{pMT1}$ ::Cm *recB268*::Tn*10* Phi80^{+}	As JJC7270 [4]
JJC7397	Δ*lacZ ycdN* ::parS$_{pMT1}$ ::FRT *araC* ::*GFP-parB*$_{pMT1}$ ::Cm *recD1901*::Tn*10* Phi80^{+}	[4]

JJC7398	Δ*lacZ ycdN* ::parS$_{pMT1}$ ::FRT *araC* ::*GFP-parB*$_{pMT1}$ ::Cm Δ*recA::*Kan Phi80^{+}	JJC7260 * P1 JJC1412
JJC7400	Δ*lacZ ydeV* ::parS$_{pMT1}$ ::FRT *araC* ::*GFP-parB*$_{pMT1}$ ::FRT *matP*::FRT *ftsK*$^{\Delta CTer}$ Cm Phi80^{+}	JJC7391 * P1 JJC1176
JJC7409	Δ*lacZ ycdN* ::parS$_{pMT1}$ ::FRT *araC* ::*GFP-parB*$_{pMT1}$ ::Cm *recD1901*::Tn*10* Δ*recA::*Kan Phi80^{+}	JJC7397 * P1 JJC1412
JJC7413	Δ*lacZ ycdN* ::parS$_{pMT1}$ ::FRT *araC* ::*GFP-parB*$_{pMT1}$ ::Cm *recB268*::Tn*10* pAM-*recBCD* Phi80^{+}	JJC7396 transformed with pAM-*recBCD*
JJC7414	Δ*lacZ ydeV* ::parS$_{pMT1}$ ::FRT *araC* ::*GFP-parB*$_{pMT1}$ ::FRT *matP*::FRT *ftsK*$^{\Delta CTer}$ Cm *recB268*::Tn*10* Phi80^{+}	JJC7400 * P1 JJC777
JJC7420	Δ*lacZ ycdN* ::parS$_{pMT1}$ ::FRT *araC* ::*GFP-parB*$_{pMT1}$ ::Cm *recB268*::Tn*10* pAM-*recBCD* Δ*recA::*Kan Phi80^{+}	JJC7413 * P1 JJC1412
JJC7441	Δ*matP::*FRT	JJC6966 excised of CmR by FRT recombination
JJC7442	Δ*lacZ ycdN* ::parS$_{pMT1}$ ::FRT *araC* ::*GFP-parB*$_{pMT1}$ ::Cm *recB268*::Tn*10* Δ*recA::*Kan Phi80^{+}	JJC7420 cured of pAM-*recBCD*
JJC7451	Δ*matP::*FRT *ftsK*$^{\Delta CTer}$ Cm	JJC7451 * P1 JJC1176
JJC7459	ΔLC3-R111 *araC* ::*GFP-parB*$_{pMT1}$ ::Apra *pspE*:: *parS* $_{pMT1}$*::*Cm Phi80^{+}	[4]
JJC7486	ΔLC3-R111 *araC* ::*GFP-parB*$_{pMT1}$ ::Apra *pspE*:: *parS* $_{pMT1}$*::*Cm Δ(*recA-srl*)::Tn*10* Phi80^{+}	JJC7459 * P1 JJC275
JJC7496	Δ*matP::*FRT *ftsK*$^{\Delta CTer}$ Cm *recB268*::Tn*10*	JJC7451 * P1 JJC777
JJC7498	Δ*lacZ ydeV* ::parS$_{pMT1}$ ::FRT *araC* ::*GFP-parB*$_{pMT1}$ ::FRT Δ*sbcD::*Kan Phi80^{+}	JJC7252 * P1 JJC1004
JJC7499	Δ*lacZ ydeV* ::parS$_{pMT1}$ ::FRT *araC* ::*GFP-parB*$_{pMT1}$ ::FRT Δ*sbcD::*Kan Δ*sbcB::*Cm Phi80^{+}	JJC7498 * P1 JJC1004
JJC7502	Δ*lacZ ydeV* ::parS$_{pMT1}$ ::FRT *araC* ::*GFP-parB*$_{pMT1}$ ::FRT Δ*sbcD::*Kan Δ*sbcB::*Cm *recB268*::Tn*10* Phi80^{+}	JJC7499 * P1 JJC777
JJC7507	*tos*::kan inserted at position 1585	MG58 [5]
JJC7508	*tos*::kan inserted at position 1585 TelN (N15)	MG59 [5]

JJC7516	Δ*lacZ yoaC* ::parS$_{pMT1}$ :: FRT *araC* ::*GFP-parB*$_{pMT1}$ ::FRT Δ*matP*::Cm Phi80^{+}	JJC7277 * P1 JJC6932
JJC7517	Δ*lacZ yoaC* ::parS$_{pMT1}$ :: FRT *araC* ::*GFP-parB*$_{pMT1}$ ::FRT Δ*sbcD*::Kan Phi80^{+}	JJC7277 * P1 JJC1004
JJC7518	*tos*::kan inserted at position 1585 *ydeV* ::parS$_{pMT1}$ Phi80^{+}	JJC7507 * P1 JJC6742
JJC7519	*tos*::kan inserted at position 1585 TelN (N15) *ydeV* ::parS$_{pMT1}$	JJC7508 * P1 JJC6742
JJC7520	*tos*::kan inserted at position 1585 TelN (N15) *recB268*::Tn*10*	JJC7508 * P1 JJC777
JJC7521	Δ*lacZ yoaC* ::parS$_{pMT1}$ :: FRT *araC* ::*GFP-parB*$_{pMT1}$ ::FRT Δ*sbcD*::Kan Δ*sbcB*::Cm *his4* Phi80^{+}	JJC7217 * P1 JJC1004
JJC7524	Δ*lacZ yoaC* ::parS$_{pMT1}$ :: FRT *araC* ::*GFP-parB*$_{pMT1}$ ::FRT Δ*matP*::FRT Phi80^{+}	JJC7516 excised of CmR by FRT recombination
JJC7525	*tos*::kan inserted at position 1585 *ydeV* ::parS$_{pMT1}$ *araC* ::*GFP-parB*$_{pMT1}$ ::Apra Phi80^{+}	JJC7518 * P1 JJC7369
JJC7526	*tos*::kan TelN (N15) *ydeV* ::parS$_{pMT1}$ *araC* ::*GFP-parB*$_{pMT1}$ ::Apra	JJC7519 * P1 JJC7369
JJC7531	Δ*lacZ ydeV* ::parS$_{pMT1}$ ::FRT *araC* ::*GFP-parB*$_{pMT1}$ ::FRT Δ*sbcD*::Kan Δ*sbcB*::Cm Δ(*recA-srl*)::Tn*10* Phi80^{+}	JJC7499 * P1 JJC275
JJC7532	*tos*::kan *ydeV* ::parS$_{pMT1}$ *araC* ::*GFP-parB*$_{pMT1}$ ::Apra Phi80^{+}	JJC7525 * P1 JJC777
JJC7533	*tos*::kan TelN (N15) *ydeV* ::parS$_{pMT1}$ *araC* ::*GFP-parB*$_{pMT1}$ ::Apra	JJC7526 * P1 JJC777
JJC7534	Δ*lacZ yoaC* ::parS$_{pMT1}$ :: FRT *araC* ::*GFP-parB*$_{pMT1}$ ::FRT Δ*sbcD*::Kan Δ*sbcB*::Cm *his4 recB268*::Tn*10* Phi80^{+}	JJC7521 * P1 JJC777
JJC7535	Δ*lacZ yoaC* ::parS$_{pMT1}$ :: FRT *araC* ::*GFP-parB*$_{pMT1}$ ::FRT Δ*matP*::FRT *recB268*::Tn*10* Phi80^{+}	JJC7524 * P1 JJC777
JJC7536	Δ*lacZ yoaC* ::parS$_{pMT1}$ :: FRT *araC* ::*GFP-parB*$_{pMT1}$ ::FRT Δ*sbcD*::Kan Δ*sbcB*::Cm *his4* Δ(*recA-srl*)::Tn*10* Phi80^{+}	JJC7521 * P1 JJC275
JJC7537	Δ*lacZ yoaC* ::parS$_{pMT1}$ :: FRT *araC* ::*GFP-parB*$_{pMT1}$ ::FRT Δ*matP*::FRT *ftsK*$^{\Delta CTer}$ Cm Phi80^{+}	JJC7524 * P1 JJC1176
JJC7544	Δ*lacZ yoaC* ::parS$_{pMT1}$ :: FRT *araC* ::*GFP-parB*$_{pMT1}$ ::FRT Δ*matP*::FRT *ftsK*$^{\Delta CTer}$ Cm *recB268*::Tn*10* Phi80^{+}	JJC7537 * P1 JJC777
JJC7552	*tos*::kan *araC* ::*GFP-parB*$_{pMT1}$ ::Apra	JJC7507 * P1 JJC7369

JJC7553	*tos*::kan TelN (N15) *araC* ::*GFP-parB*$_{pMT1}$ ::Apra	JJC7508 * P1 JJC7369
JJC7555	*tos*::kan *pspE*:: *parS* $_{pMT1}$::Cm	JJC7507 * P1 JJC7457
JJC7556	*tos*::kan TelN (N15) *pspE*:: *parS* $_{pMT1}$::Cm	JJC7508 * P1 JJC7457
JJC7557	*tos*::kan TelN (N15) *gusC*:: *parS* $_{pMT1}$::Cm *yzeB*::parB$_{P1}$-trim	F. Boccard laboratory collection
JJC7559	*tos*::kan *pspE*:: *parS* $_{pMT1}$::Cm *araC* ::*GFP-parB*$_{pMT1}$ ::Apra	JJC7555 *P1 JJC7369
JJC7560	*tos*::kan TelN (N15) *pspE*:: *parS* $_{pMT1}$::Cm *araC* ::*GFP-parB*$_{pMT1}$ ::Apra	JJC7556 *P1 JJC7369
JJC7563	*tos*::kan TelN (N15) *gusC*:: *parS* $_{pMT1}$::Cm *yzeB*::parB$_{P1}$-trim *araC* ::*GFP-parB*$_{pMT1}$ ::Apra	JJC7557 * P1 JJC7369
JJC7569	*tos*::kan *pspE*:: *parS* $_{pMT1}$::Cm *araC* ::*GFP-parB*$_{pMT1}$ ::Apra *recB268*::Tn*10*	JJC7559 * P1 JJC777
JJC7570	*tos*::kan TelN (N15) *pspE*:: *parS* $_{pMT1}$::Cm *araC* ::*GFP-parB*$_{pMT1}$ ::Apra *recB268*::Tn*10*	JJC7560 * P1 JJC777
JJC7571	*tos*::kan TelN (N15) *gusC*:: *parS* $_{pMT1}$::Cm *yzeB*::parB$_{P1}$trim *araC* ::*GFP-parB*$_{pMT1}$ ::Apra *recB268*::Tn*10*	JJC7563 * P1 JJC777
JJC7588	DY330 *yddW*:: *parS* $_{pMT1}$::Cm	Gene replacement of the *yddW* gene by a PCR fragment carrying *parS* $_{pMT1}$ and the CmR marker
JJC7590	*tos*::kan *araC* ::*GFP-parB*$_{pMT1}$ ::Apra *yddW*:: *parS* $_{pMT1}$::Cm	JJC7552 * P1 JJC7588
JJC7591	*tos*::kan TelN (N15) *araC* ::*GFP-parB*$_{pMT1}$ ::Apra *yddW*:: *parS* $_{pMT1}$::Cm	JJC7553 * P1 JJC7588
JJC7594	*tos*::kan *araC* ::*GFP-parB*$_{pMT1}$ ::Apra *yddW*:: *parS* $_{pMT1}$::Cm *recB268*::Tn*10*	JJC7590 * P1 JJC777
JJC7595	*tos*::kan TelN (N15) *araC* ::*GFP-parB*$_{pMT1}$ ::Apra *yddW*:: *parS* $_{pMT1}$::Cm *recB268*::Tn*10*	JJC7591 * P1 JJC777

1. Draper GC, McLennan N, Begg K, Masters M, Donachie WD (1998) Only the N-terminal domain of FtsK functions in cell division. J Bacteriol 180: 4621-4627.
2. Corre J, Cornet F, Patte J, Louarn JM (1997) Unraveling a region-specific hyper-recombination phenomenon: Genetic control and modalities of terminal recombination in Escherichia coli. Genetics 147: 979-989.
3. Yu D, Ellis HM, Lee EC, Jenkins NA, Copeland NG, et al. (2000) An efficient recombination system for chromosome engineering in Escherichia coli. Proc Natl Acad Sci U S A 97: 5978-5983.
4. Sinha AK, Durand A, Desfontaines JM, Iurchenko I, Auger H, et al. (2017) Division-induced DNA double strand breaks in the chromosome terminus region of Escherichia coli lacking RecBCD DNA repair enzyme. PLoS Genet 13: e1006895.
5. Cui T, Moro-oka N, Ohsumi K, Kodama K, Ohshima T, et al. (2007) Escherichia coli with a linear genome. EMBO Rep 8: 181-187.
