# Supplementary material for: Broken replication forks trigger heritable DNA breaks in the terminus of a circular chromosome
Source: PLoS Genet. 2018 Mar 9;14(3):e1007256. doi: 10.1371/journal.pgen.1007256 (PMC5862497; doi:10.1371/journal.pgen.1007256)
Supplement: S2 Table — (PDF) [file pgen.1007256.s002.pdf]

S2 Table Percentages of cells with zero, one or two foci in different mutants.

***ydeV*::*parS*$_{pMT1}$**	% of cells				N (n)
Foci per cells	0	1	2	>2	
wild-type[(a)]	0.6 ± 0.2	81.2 ± 2.1	18.1 ± 2.3	0.12 ± 0.1	1547 (3)
recB[(a)]	32 ± 1.5	57.2 ± 2.2	10.1 ± 1.9	0.7 ± 0.15	1397 (3)
recC[(a)]	30.6 ± 1.6	59.2 ± 3.9	8.7 ± 4.9	1.5 ± 0.7	866 (2)
recA	9 ± 2.8	76.4 ± 3	14.3 ± 1.6	0.3 ± 0.4	2545 (6)
recD	0.6 ± 0.7	82.9 ± 1.2	16.3 ± 1.5	0.17 ± 0.3	1184 (3)
recA recB	36.6 ± 1.5	53.7 ± 2.9	8.9 ± 2.2	0.77 ± 0.23	1643 (3)
recA recC	36.4 ± 1.6	52.3 ± 08	11 ± 0.8	0.2 ± 0.01	1258 (3)
recA recD	27.3 ± 2.1	61.5 ± 3.1	11.1 ± 1.6	0 ± 0	1363 (3)
sbcB sbcD	1 ± 0.1	85 ± 1.5	14 ± 1.3	0	508 (2)
sbcB sbcD recA	31 ± 1.2	62.4 ± 1.6	4.8 ± 3.4	0.07 ± 0.1	1792 (3)
recA sbcB	16.9 ± 4.6	70 ± 3.7	13.2 ± 2.3	0	1533 (3)
recA sbcD	15.3 ± 1.6	71.1 ± 2.9	13.5 ± 3	0	1576 (3)
sbcB sbcD recB	29.6 ± 2.2	59 ± 4.7	11 ± 2.2	0.7 ± 0.7	1170 (3)
ruvAB	6.8 ± 1.1	71.2 ± 2.3	18.5 ± 3.8	3.5 ± 1.3	1268 (3)
ruvAB recB	37 ± 2.1	50.7 ± 0.7	10.5 ± 0.8	1.8 ± 1	1476 (3)
ruvAB recA	11.7 ± 0.7	69.1 ± 4.1	19 ± 3.3	0.1 ± 0.1	1016 (2)
ruvAB recA recB	37.6 ± 2.2	50 ± 3.8	12.5 ± 1.7	0	1511 (3)
recA tus	16.5 ± 0.9	64.9 ± 5.3	18.2 ± 4.5	0.4 ± 0.3	1595 (3)
matP	1 ± 0.9	61.2 ± 7.1	37.6 ± 7.3	0.2 ± 0.3	1029 (3)
matP recB	37.6 ± 2	44 ± 5.6	17 ± 4.8	1.5 ± 0.3	1306 (3)
ftsK$^{\Delta Cter}$ *matP*[(b)]	14.6 ±2.1	39 ± 7.5	33.5 ± 7.8	12.9 ± 1.7	1057 (3)
ftsK$^{\Delta Cter}$ *matP recB*	39.7 ± 1.2	27 ± 1.9	17 ± 3.3	6.3 ± 1	1028 (3)
Circular *tos*	1.8 ± 0.7	78.5 ± 2.4	19.6 ± 1.7	0.08 ± 0.11	1240 (2)
Circular *tos recB*	31.9 ± 2	52.6 ± 3.1	15.4 ± 5.2	0.11 ± 0.19	1882 (2)
Linear *tos*	4.8 ± 0.14	76.3 ± 1.2	18.6 ± 0.7	0.23 ± 0.32	1045 (2)
Linear *tos recB*	20.7 ± 1.4	59.4 ± 4.3	18.4 ± 2.5	0.91 ± 0.4	1534 (2)

***yoaC::parS*_pMT1**	% of cells				N (n)
Foci per cells	0	1	2	>2	
wt[(a)]	0.6 ± 0.3	58.4 ± 1.1	40.7 ± 1.6	0.2 ± 0.19	1491 (3)
recB[(a)]	7.9 ± 1	51.3 ± 1.1	38 ± 1.4	2.8 ± 0.9	1420 (3)
recA	8.8 ± 0.9	64.6 ± 2.7	1.6 ± 1.6	0.14 ± 0.2	1254 (2)
recD	0.34 ± 0.37	59.1 ± 1.5	40.2 ± 2.1	0.37 ± 0.6	1030 (3)
recA recB	8.5 ± 1.6	57.8 ± 2.5	32.5 ± 3	1.3 ± 0.3	1631 (3)
recA recD	23 ± 2.1	54 ± 1.5	23.5 ± 3.8	1 ± 0.3	695 (2)
sbcB sbcD	1.7 ± 0.8	67.1 ± 6.2	31 ± 5.4	0.22 ± 0.02	950 (2)
sbcB sbcD recA	11.6 ± 1	64.9 ± 1.7	22.6 ± 2.7	0.8 ± 0.01	736 (2)
sbcB sbcD recB	5.9 ± 0.2	65.7 ± 1.5	26.5 ± 1.6	1.9 ± 0.08	858 (2)
matP	1.4 ± 0.06	65.8 ± 4.7	32.5 ± 4.7	0.34 ± 0.01	583 (2)
matP recB	9.2 ± 1.5	56.9 ± 1.8	31.2 ± 3.2	2.7 ± 0.06	822 (2)

***ycdN::parS*_pMT1**	% of cells				N
Foci per cells	0	1	2	>2	
wt[(a)]	0.95 ± 0.88	59.2 ± 3.2	39.7 ± 3.9	0.17 ± 0.2	684 (2)
recB[(a)]	6.7 ± 0.17	55.5 ± 1.1	36.2 ± 0	1.6 ± 0.95	625 (2)
recA	5.2 ± 0.2	63.6 ± 4.3	31.2 ± 4.1	0 ± 0	602 (2)
recA recB	5 ± 0.2	51.2 ± 4.5	41.1 ± 3.5	2.7 ± 0.8	648 (2)
recD	0.5 ± 0.04	55.7 ± 1.4	43 ± 1	0.84 ± 0.45	1246 (2)
recA recD	11.4 ± 1.1	47.6 ± 1.5	38.3 ± 1.9	2.15 ± 0.7	747 (2)

***pspE::parS*_pMT1**	% of cells				N
Foci per cells	0	1	2	>2	
Circular *tos*	1.4 ± 0.3	59.7 ± 1.6	38.6 ± 1.2	0.2 ± 0.3	946 (2)
Circular *tos recB*	15.3 ± 3.2	49.5 ± 0.2	33.6 ± 3.3	1.6 ± 0.4	945 (2)
Linear *tos*	1.1 ± 0.01	62.2 ± 4.4	35.9 ± 3.2	0.75 ± 1	893 (2)
Linear *tos recB*	56.4 ± 1.6	26.2 ± 0.4	17.1 ± 1.5	0.1 ± 0.14	911 (2)

***yddW*::*parS*$_{pMT1}$**	% of cells				N (n)
Foci per cells	0	1	2	>2	
Circular *tos*	1.4 ± 0.7	82.4 ± 1.7	16.2 ± 2.4	0	1190 (2)
Circular *tos recB*	29.6 ± 2.6	46.9 ± 2.2	22.4 ± 4.8	1.1 ± 0.01	1015 (2)
Linear *tos*	4.1 ± 2.8	75.7 ± 2.5	20.6 ± 0.2	0.5 ± 0.1	1051 (2)
Linear *tos recB*	59.6 ± 6.2	32.4 ± 2.5	7.3 ± 2.7	0.65 ± 0.9	1123 (2)

***gus*::*parS*$_{pMT1}$**	% of cells				N (n)
Foci per cells	0	1	2	>2	
Linear *tos*	2.4 ± 0.02	74.3 ± 3.3	22.8 ± 3.9	0.4 ± 0.6	1047 (2)
Linear *tos recB*	10.5 ± 0.9	64.7 ± 3.8	23.3 ± 5.5	1.5 ± 0.8	995 (2)

"% cells" shows the averages of two or three independent experiments ± standard deviation.
N = number of cells analyzed. (n) number of independent experiments
(a) published in [1]
(b) *ftsK*$^{\Delta Cter}$ *matP* are very elongated

1 . Sinha AK, Durand A, Desfontaines JM, Iurchenko I, Auger H, et al. (2017) Division-induced DNA double strand breaks in the chromosome terminus region of Escherichia coli lacking RecBCD DNA repair enzyme. PLoS Genet 13: e1006895.
