# Supplementary material for: Broken replication forks trigger heritable DNA breaks in the terminus of a circular chromosome
Source: PLoS Genet. 2018 Mar 9;14(3):e1007256. doi: 10.1371/journal.pgen.1007256 (PMC5862497; doi:10.1371/journal.pgen.1007256)
Supplement: S3 Table — (PDF) [file pgen.1007256.s003.pdf]

S3 Table : Oligonucleotides used in this study.

608<br><br>609	AAGTCGCTGGGGCAGTTAAACTCCCATTCTTACTTTGTAA CAAGCCGGTGGTGTAGGCTGGAGCTGCTTC<br>CATGATTATTAGTGGGATAGTTTAAGAGGGTAACAAGCC GGTGGGTAAAGCTTACCCGTCTTACTGTCGG	Primers to insert *parS*$_{pMT1}$ in the intergenic region between *yddW* and *yddV*
610<br>611	AGCGGCACCATCTTGTTCCG<br>ACAAGTCCGGTCCACTCATC	Primers to verify yddW::parSpMT1
604<br>605	GATTTTCTTACAGGTGTAGGC<br>TCAGAAGTCCGTCAATCCGGG	Primers to verify tos::kanR
