# Supplementary material for: Broken replication forks trigger heritable DNA breaks in the terminus of a circular chromosome
Source: PLoS Genet. 2018 Mar 9;14(3):e1007256. doi: 10.1371/journal.pgen.1007256 (PMC5862497; doi:10.1371/journal.pgen.1007256)
Supplement: S4 Table — (PDF) [file pgen.1007256.s004.pdf]

Table S4: Percentage of cells with zero, one or two foci, ratio of initial events and of inherited events are independent of strain background

ydeV::parS$_{\mathbf{pMT1}}$	% of cells				N (n)	Initial events	transmitted
Foci per cells	0	1	2	>2			
wild-type Phi-	1.1 ± 0.4	83 ± 2.6	16.1 ± 2.7	0.1 ± 0.1	1158 (3)		
recB Phi-	41.7 ± 7.7	47.1 ± 7.6	10.2 ± 2.4	1 ± 0.9	1813 (3)	19% (791)	80.2%
recA Phi-	8.9 ± 1.6	77.4 ± 3.3	13.3 ± 3.1	0.4 ± 0.3	1751 (3)	4.9% (610)	35.4%
