# Supplementary figures and images for: Broken replication forks trigger heritable DNA breaks in the terminus of a circular chromosome
Source: PLoS Genet. 2018 Mar 9;14(3):e1007256. doi: 10.1371/journal.pgen.1007256 (PMC5862497; doi:10.1371/journal.pgen.1007256)

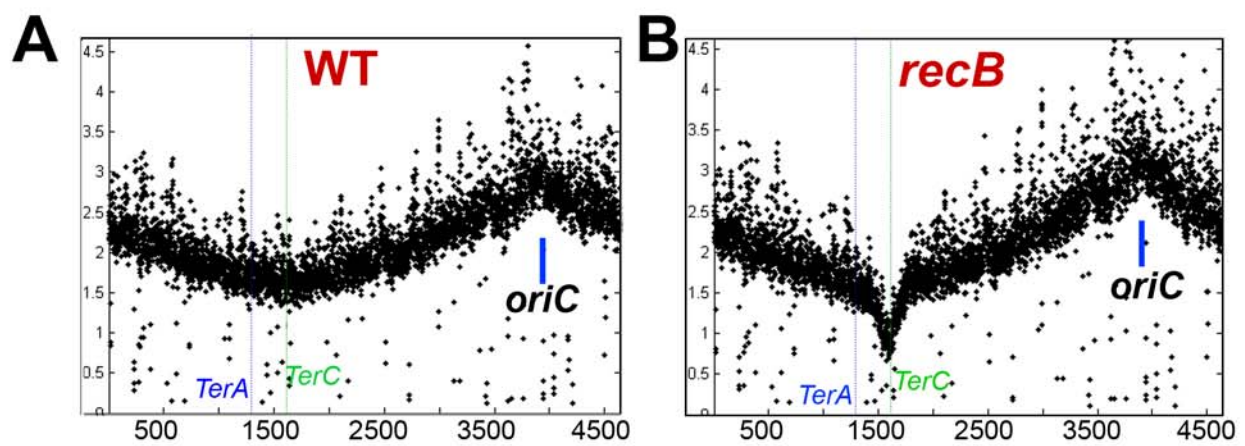

Figure supplement 1  
Marker frequency analysis of wild-type and *recB* mutant.

Supplement: S1 Fig — (A) wild-type. (B) recB mutant. Normalized replication profiles of exponentially growing cells are shown. Sequence read frequencies are normalized to the total number of reads and then the normalized reads (y-axis) are plotted against the chromosome coordinates in kb (x-axis). The approximate position of replication termination sites terA and terC and oriC are marked in each plot. (PDF) [file pgen.1007256.s005.pdf]

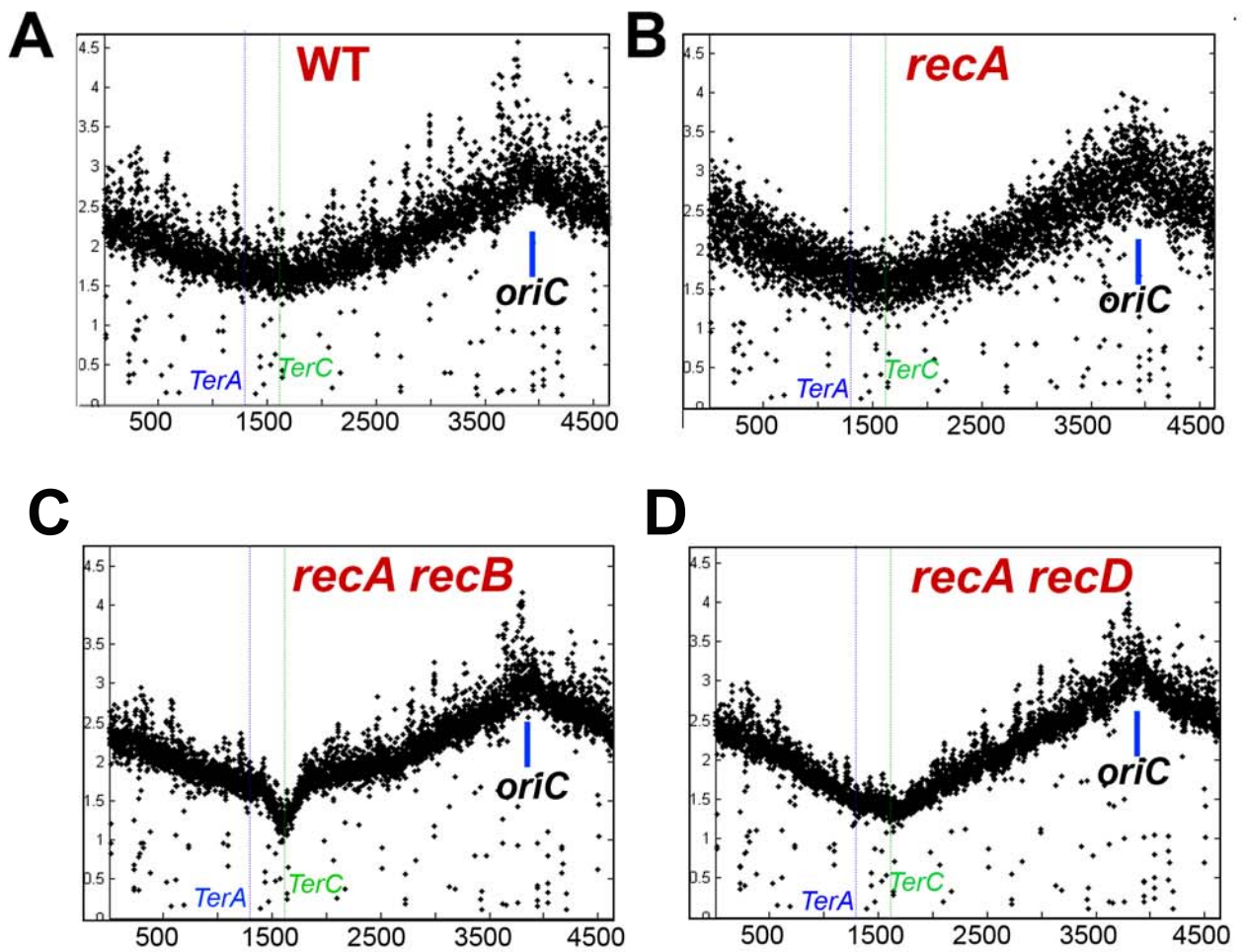

Figure Supplement 2

Marker frequency analysis of *recA*, *recA recB* and *recA recD* mutants

Supplement: S2 Fig — (A) wild-type, (B) recA, (C) recA recB and (D) recA recD mutants. See legend of S1 Fig. (PDF) [file pgen.1007256.s006.pdf]

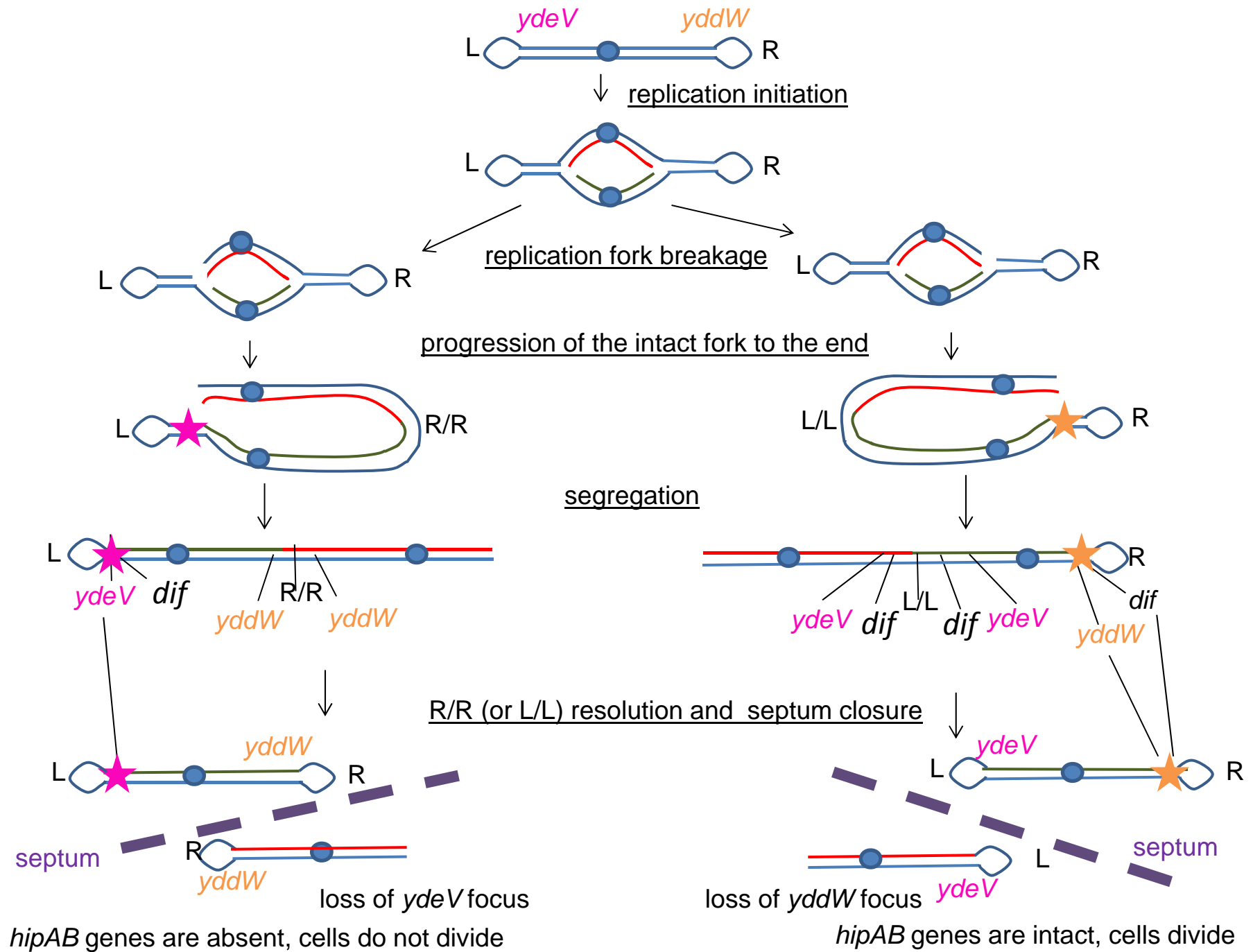

Supplement: S6 Fig — In a first step, during replication progression one replication fork is accidentally broken. On the left part of the figure the left fork is broken, and on the right part of the figure the right fork is broken. The other replication fork progresses to the end of the chromosome, generating a linear dimer with an inverted duplication of the replicated right (or left) tos hairpin (Tel R/R (R/R), or Tel L/L (L/L) regions [63]). The replication origins segregate to the two cell halves and because the Tel R/R and Tel L/L regions are regions of KOPS convergence and MatP binding, they localize in the middle of the cell, where the septum forms. Resolution of the tos sites by TelN [63] creates an intact linear chromosome and a partial one that lacks all non-replicated chromosome sequences between the initial replication fork break and the terminus. The daughter cell that inherits the intact linear chromosome shows a focus and propagates normally. The one that carries the partial chromosome lacks the yddW::parSpMT1 or ydeV::parSpMT1 site, depending on the position of the initial DSB. In cells that lack yddW::parSpMT1 the hipA hipB genes are intact, and cells can multiply until they lack some essential protein. In cells that lack ydeV::parS pMT1 the hipA hipB genes are absent, and growth is prevented by the long-lived HipA protein. Blue lines, initial chromosome DNA strands; red and green lines, newly synthesized DNA strands; blue circles, replication origins; stars, yddW::parSpMT1 (yellow) or ydeV::parSpMT1 (pink) sites; dashed purple line, septum. L and R indicate the left and right tos hairpins, LL/ and R/R the inversely duplicated sites after replication. The position of the dif site is also indicated. (PDF) [file pgen.1007256.s010.pdf]
