# Supplementary material for: Broken replication forks trigger heritable DNA breaks in the terminus of a circular chromosome
Source: PLoS Genet. 2018 Mar 9;14(3):e1007256. doi: 10.1371/journal.pgen.1007256 (PMC5862497; doi:10.1371/journal.pgen.1007256)
Supplement: S3 Fig — (A) ruvAB, (B) ruvAB recB, (C) sbcB sbcD, and (D) recA sbcB sbcD mutants. See legend of S1 Fig. (PDF) [file pgen.1007256.s007.pdf]

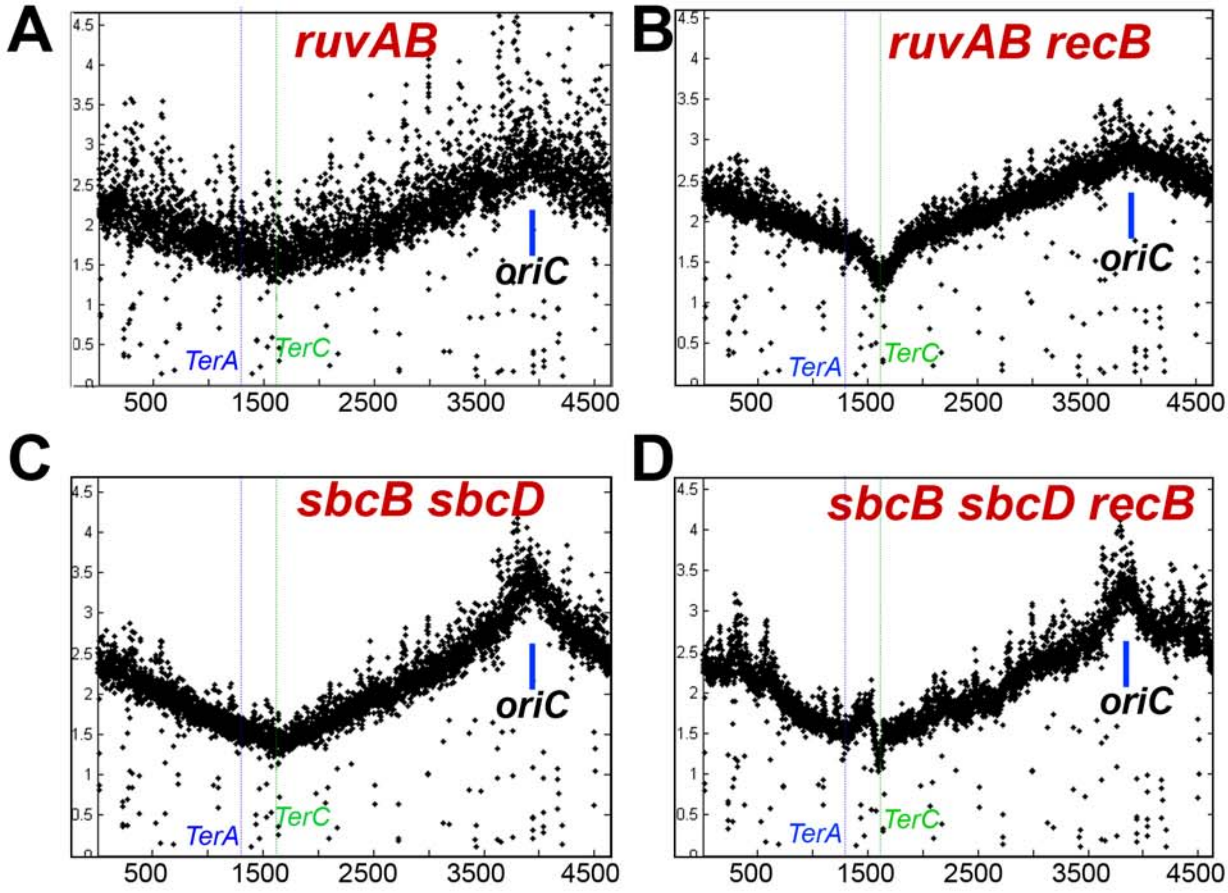

Figure Supplement 3
Marker frequency analysis of *ruvAB, ruvAB recB*, *sbcB sbcD* and *sbcB sbcD recB* mutants
