# Supplementary material for: Broken replication forks trigger heritable DNA breaks in the terminus of a circular chromosome
Source: PLoS Genet. 2018 Mar 9;14(3):e1007256. doi: 10.1371/journal.pgen.1007256 (PMC5862497; doi:10.1371/journal.pgen.1007256)
Supplement: S4 Fig — See legend of S1 Fig. (PDF) [file pgen.1007256.s008.pdf]

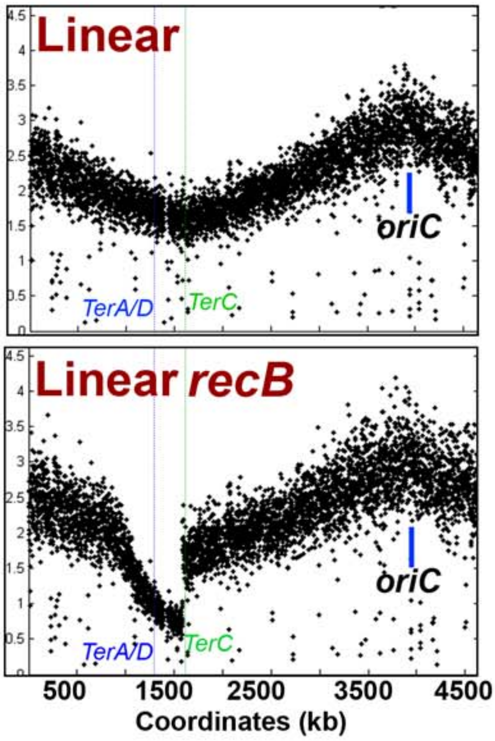

Figure Supplement 4
Marker frequency anaysis of wild-type and *recB* mutants with a linear chromosome
