# Supplementary material for: Broken replication forks trigger heritable DNA breaks in the terminus of a circular chromosome
Source: PLoS Genet. 2018 Mar 9;14(3):e1007256. doi: 10.1371/journal.pgen.1007256 (PMC5862497; doi:10.1371/journal.pgen.1007256)
Supplement: S5 Fig — (A) matP, (B) matP recB, (C) matP ftsKΔCTer and (D) matP ftsKΔCTer recB mutants. See legend of S1 Fig. (PDF) [file pgen.1007256.s009.pdf]

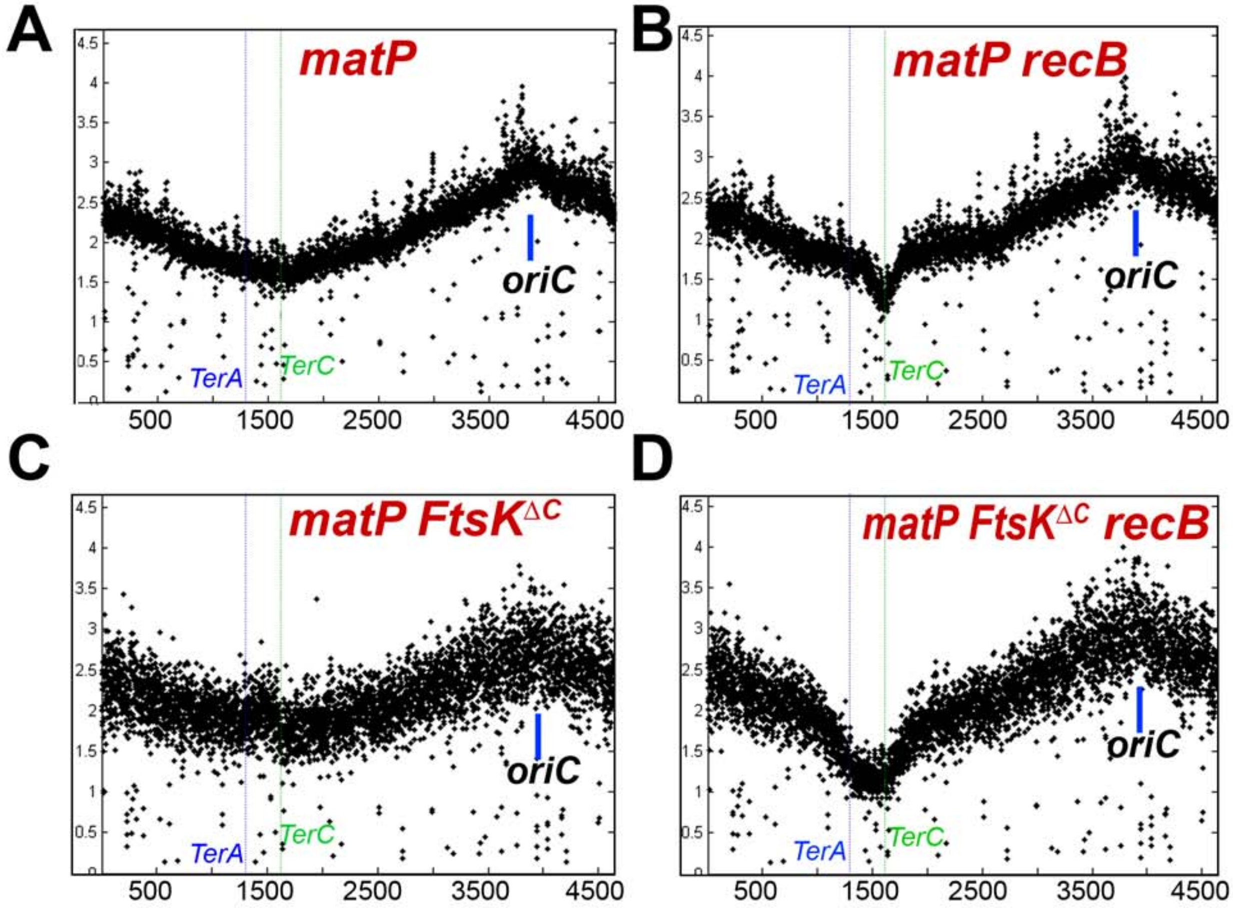

Figure Supplement 5
Marker frequency analysis of *matP*, *matP recB*, *matP FtsK* and *matP ftsK recB* mutants
